# A dual insect symbiont and plant pathogen improves insect host fitness under arginine limitation

**DOI:** 10.1128/mbio.03588-24

**Published:** 2025-02-25

**Authors:** Younghwan Kwak, Jacob A. Argandona, Sen Miao, Thomas J. Son, Allison K. Hansen

**Affiliations:** 1 Department of Life and Environmental Sciences, University of California, Merced, California, USA; 2 Department of Entomology, University of California, Riverside, California, USA; Max Planck Institute for Chemical Ecology, Jena, Germany

**Keywords:** facultative symbiont, nutritional benefit, argininosuccinate lyase, arginine, horizontal gene transfer, *Liberibacter psyllaurous*, *Bactericera cockerelli*, *Carsonella*, *Wolbachia*, toll pathway

## Abstract

Some facultative bacterial symbionts are known to benefit insects, but nutritional advantages are rare among these non-obligate symbionts. Here, we demonstrate that the facultative symbiont *Candidatus Liberibacter psyllaurous* enhances the fitness of its psyllid insect host, *Bactericera cockerelli*, by providing nutritional benefits. *L. psyllaurous*, an unculturable pathogen of solanaceous crops, also establishes a close relationship with its insect vector, *B. cockerelli*, increasing in titer during insect development, vertically transmitting through eggs, and colonizing various tissues, including the bacteriome, which houses the obligate nutritional symbiont, *Carsonella. Carsonella* supplies essential amino acids to its insect host but has gaps in some of its essential amino acid pathways that the psyllid complements with its own genes, many of which have been acquired through horizontal gene transfer (HGT) from bacteria. Our findings reveal that *L. psyllaurous* increases psyllid fitness on plants by reducing developmental time and increasing adult weight. In addition, through metagenomic sequencing, we reveal that *L. psyllaurous* maintains complete pathways for synthesizing the essential amino acids arginine, lysine, and threonine, unlike the psyllid’s other resident microbiota, *Carsonella,* and two co-occurring *Wolbachia* strains. RNA sequencing reveals the downregulation of a HGT collaborative psyllid gene (*ASL*), which indicates a reduced demand for arginine supplied by *Carsonella* when the psyllid is infected with *L. psyllaurous*. Notably, artificial diet assays show that *L. psyllaurous* enhances psyllid fitness on an arginine-deplete diet. These results corroborate the role of *L. psyllaurous* as a beneficial insect symbiont, contributing to the nutrition of its insect host.

**IMPORTANCE:**

Unlike obligate symbionts that are permanently associated with their hosts, facultative symbionts rarely show direct nutritional contributions, especially under nutrient-limited conditions. This study demonstrates, for the first time, that *Candidatus Liberibacter psyllaurous*, a facultative symbiont and a plant pathogen, enhances the fitness of its *Bactericera cockerelli* host by supplying an essential nutrient arginine that is lacking in the plant sap diet. Our findings reveal how facultative symbionts can play a vital role in helping their insect hosts adapt to nutrient-limited environments. This work provides new insights into the dynamic interactions between insect hosts, their symbiotic microbes, and their shared ecological niches, broadening our understanding of symbiosis and its role in shaping adaptation and survival.

## INTRODUCTION

Many insects harbor facultative symbionts—microbes that, although not essential for survival, can significantly impact the ecology and evolution of their insect hosts (1, 2). Under various ecological conditions, these symbionts can enhance insect host fitness by conferring resistance to natural enemies, heat stress, insecticides, or pathogens (3–9). Some facultative symbionts take even more complex roles by functioning as both bacterial insect symbionts and plant pathogens, thereby bridging two distinct host environments and ecologies (10–13). The dual roles of these facultative symbionts—as mutualistic or commensal partners in insect vectors and as pathogens in plants—pose intriguing questions about how they navigate between such contrasting environments and relationships. Despite their versatility across various host environments, the interactions between these facultative symbionts, insect hosts, and their resident microbiota remain largely understudied.

Dual facultative symbionts of both plants and insects are primarily observed in sap-feeding insects. These insects, often classified as pests, transmit and interact with microbes that can colonize the plant’s vascular system through their specialized feeding habits (14). However, many of these bacterial plant pathogens are not passive travelers in their insect hosts; some in fact complete their lifecycle within insects, colonize tissues, persist across the insect host’s lifespan and can be vertically transmitted to the next generation (12, 14, 15). Interestingly, several of these bacterial symbionts such as *Rickettsia*, *Arsenophonus-*like, and *Phytoplasma*, which are also known as plant pathogens, evolved from lineages of arthropod symbionts rather than plant endophytes (16–19). Some dual facultative symbionts have also been observed to increase their insect host’s fitness. For example, some *Phytoplasma* species, which cause aster yellows and Witches’ Broom, improve leafhopper survival and reproduction (20, 21). Several studies have also reported that the serious bacterial plant pathogen of citrus, *Candidatus Liberibacter asiaticus*, significantly increases the fecundity of the psyllid, *Diaphorina citri* (22–24). These intricate relationships emphasize the evolutionary adaptability and diverse impacts facultative symbionts can have in their plant versus insect hosts.

One group of sap-feeding insects, psyllids (Sternorrhyncha: Psylloidea), are known to harbor a diversity of heritable symbionts, encompassing both obligate and facultative associations (25–29). The obligate symbiont “Candidatus *Carsonella ruddii,*” hereafter referred to as *Carsonella*, is a well-documented example of a nutritional symbiosis where *Carsonella* synthesizes essential amino acids for its psyllid host to survive on a nitrogen-poor diet of plant sap (30). Due to extreme genome reduction, *Carsonella* lacks genes for some essential amino acid pathways (31), and the insect host is hypothesized to compensate for some of *Carsonella’s* gene losses through the upregulation of psyllid-encoded genes (32, 33). Some of these compensatory genes in the psyllid’s genome have been acquired through horizontal gene transfer (HGT) from bacteria to an early ancestor of psyllids, suggesting that these genes play a crucial role in the psyllid-nutritional symbioses (32–34).

Alongside *Carsonella*, other symbionts have formed long-term evolutionary associations with their psyllid hosts. This is demonstrated by their consistent presence within psyllid species, their highly reduced genomes, and their co-speciation with related psyllid lineages (25–27, 35–38). These symbionts are hypothesized to be transitioning toward an obligate role by providing the psyllid with defensive capabilities and/or complementing *Carsonella*’s incomplete essential amino acid and vitamin biosynthesis pathways (36–40). In addition to the long-term co-symbionts that are fixed within a psyllid species and may complement *Carsonella*, facultative symbionts are also present, spanning a variety of bacterial taxa. Most of our understanding of psyllid facultative symbionts is based on their identity and presence and absence in psyllid species and individuals based on 16S rRNA sequencing surveys (4, 25–27, 29, 37, 41). One of the most widespread and common bacterial taxa detected in psyllid species to date based on 16S rRNA high-throughput sequencing analyses is *Wolbachia* (26, 27, 35, 41, 42). The 16S rRNA high-throughput screening of psyllid species has also been recently used to detect novel *Candidatus Liberibacter* species (27). A total of 10 *Candidatus Liberibacter* species have been identified to date, and a few of these species are known to be both significant plant pathogens vectored by psyllids and facultative symbionts of these psyllid vectors (43).

*Candidatus Liberibacter psyllaurous* (also known as *Ca*. *L. solanacearum*; hereafter referred to as *L. psyllaurous*) exemplifies this dual functionality, acting both as a facultative symbiont in the psyllid *Bactericera cockerelli* and as an important economic plant pathogen in solanaceous crops, especially potato (11, 44, 45). Although much of the research on this bacterium has focused on its pathogenicity in plants, its role with its psyllid host and its resident microbes remains less explored. In psyllids, this bacterium is vertically transmitted in eggs and maintains high genome copy numbers as the psyllid develops (46). The symbiont’s reduced genome (approximately 1.2 Mb) indicates a high degree of metabolic dependence on its hosts (47), a trait common among many non-culturable facultative symbionts (48). Nevertheless, the possibility that *L. psyllaurous* contributes metabolically to its psyllid host cannot be entirely disregarded, especially given its persistence in insect host tissues and the documented fitness benefits conferred by related species (22–24).

In the psyllid host, *L. psyllaurous* can colonize a range of insect host tissues, including the bacteriome (49), a specialized insect organ that houses *Carsonella* (50). Within the *bacteriome, L. psyllaurous* may interact with *Carsonella*, potentially influencing *Carsonella*’s provision of essential amino acids to the psyllid host. In the psyllid *B. cockerelli*, *Wolbachia* is also found in psyllid bacteriomes alongside *L. psyllaurous* and *Carsonella* (32). Its presence varies between *B. cockerelli* biotypes in the U.S., and this variation may influence *L. psyllaurous* acquisition and transmission (51, 52). For instance, the Northwestern biotype of psyllids is *Wolbachia*-free and are less effective vectors for *L. psyllaurous* compared to the Central and Western biotypes that harbor *Wolbachia* (53). Notably, in another psyllid-*Liberibacter* system, *Wolbachia* was shown to repress phage lytic cycle genes in *L. asiaticus*, possibly enhancing the survival of both symbionts in their insect host *D. citri* (54). However, the genome of the *Wolbachia* species in *B. cockerelli* has yet to be sequenced, leaving many open questions about its precise role in mediating these symbiotic interactions, in addition to potential nutritional interactions with *L. psyllaurous, Carsonella,* and the insect host.

In this study, we explore how *L. psyllaurous* affects both the fitness and the resident microbiota of *B. cockerelli*. Since all psyllid symbionts, including *L. psyllaurous* are unculturable, we employ an integrative approach that combines genomic, transcriptomic, and bioassay techniques to address key questions. First, we quantify the genome copy numbers of *L. psyllaurous* and *Wolbachia* in bacteriomes and the rest of body tissues to investigate its potential interactions with the psyllid host and its symbiotic community. Second, we conduct metagenomic sequencing to reconstruct the metabolic capabilities of these symbionts, focusing on essential amino acid biosynthesis pathways that may be incomplete or absent in the psyllid’s resident microbiota. Third, taking advantage of our chromosomal-level genome of *B. cockerelli* (34), we perform RNA-seq analyses to assess how *L. psyllaurous* affects the expression of psyllid genes that collaborate with *Carsonella* for the biosynthesis of essential amino acids and vitamins, as well as psyllid genes associated with immune responses. Fourth, we conduct bioassays to evaluate *L. psyllaurous’* effects on psyllid survival and reproduction. Finally, we perform manipulated artificial diet bioassays to determine if *L. psyllaurous* can improve psyllid fitness when certain amino acids are limited from its psyllid host diet, independent of altered plant nutrients. Together, these results corroborate a nutritional mechanism by which *L. psyllaurous* can enhance psyllid host fitness.

## RESULTS

### *L. psyllaurous* and *Wolbachia* are abundant in the bacteriome with *Carsonella*

Previous studies have shown that *L. psyllaurous* and *Wolbachia* are present in bacteriomes of *B. cockerelli* (32, 49). However, their relative abundance to one another and in different tissue types remains uncertain. To investigate this, we assessed whether the presence or absence of *L. psyllaurous* affects the abundance of *Wolbachia* in bacteriomes and non-bacteriome tissues. Furthermore, we examined whether the two strains of *Wolbachia* (referred to here as *Wolbachia*-Bin1 and Bin2) display different abundances, as both strains have been observed to coexist within a single individual in field populations (52, 55).

**Fig 1:**
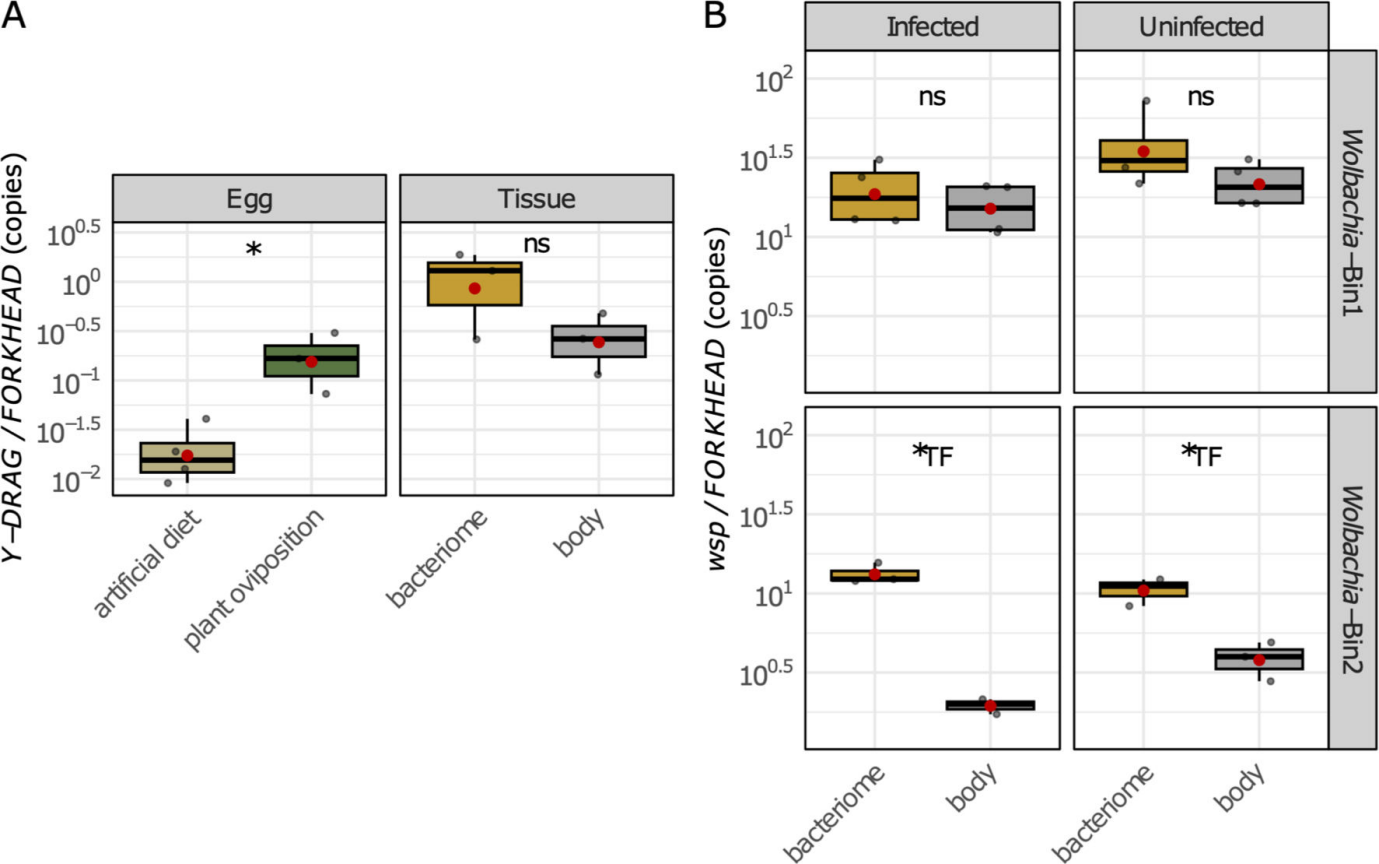
Quantification of *L. psyllaurous* and *Wolbachia* genome copy numbers in eggs, bacteriome, and body tissues of *B. cockerelli*. (*) indicates significance at *P* < 0.05. “ns” denotes non-significant differences (*P* > 0.05). Bacteriome and body tissues for each biological replicate are pooled samples from 15 fifth-instar nymphs. For egg samples, each biological replicate consists of five pooled eggs. Red dots represent means. (**A**) Normalized *L. psyllaurous* (*Y-DRAG*) to psyllid (*FORKHEAD*) gene copy numbers in egg samples from artificial diet and plant oviposition trials (left) and bacteriome and body tissues (right). Statistical significance between conditions was determined by *t*-tests (**B**) Normalized *Wolbachia* Bin1 and Bin2 (*wsp*) to psyllid (*FORKHEAD*) gene copy numbers in bacteriome and body tissues across *L. psyllaurous*-infected and uninfected tissue conditions. Statistical significance between conditions was determined by two-factor ANOVA (tissue type X *L. psyllaurous* infection status). *LF* indicates a significant effect of *L. psyllaurous* infection factor, and *TF* indicates a significant effect of tissue factor.

In *L. psyllaurous*-infected psyllids, we found that the genome copies of *L. psyllaurous* were not significantly different between bacteriome and non-bacteriome tissues (*t*-test; df = 2, *P* = 0.18; Fig. 1A). Similarly, the copy number of *Wolbachia*-Bin1 was not significantly different between bacteriome and body tissues (F_1,12_=2.461, *P* = 0.14), or influenced by the infection status of *L. psyllaurous* (F_1,12_=3.707; *P* = 0.08). There was also no significant interaction between tissue type and *L. psyllaurous* infection status for *Wolbachia*-Bin1 (F_1,12_=0.869; *P* = 0.37; Fig. 1B).

In contrast, *Wolbachia*-Bin2 exhibited significantly higher genome copy numbers in bacteriomes compared with other body tissues (F_1,12_=104.393; *P* = 7.23e-06), indicating a preferential association with the bacteriome (Fig. 1B). However, there was no significant difference in *Wolbachia*-Bin2 copies between *L. psyllaurous*-infected and uninfected tissues (F_1,12_=0.210; *P* = 0.66), although a significant interaction was detected between tissue type and *L. psyllaurous* infection status (F_1,12_=7.03; *P* = 0.03).

### Metagenome assemblies and symbiont metabolic contributions

Considering that *Wolbachia* strains and *L. psyllaurous* are abundant in the bacteriome (Fig. 1), we investigated whether *L. psyllaurous* and *Wolbachia* have the metabolic potential, alongside *Carsonella*, to contribute to the biosynthesis of essential amino acids and vitamins for the psyllid host. To explore potential metabolic interactions among these symbionts and the host, we sequenced the metagenomes of *B. cockerelli*. Our metagenomic sequencing of *B. cockerelli* symbionts yielded a complete circular assembly for *Carsonella,* and near-complete assemblies of *L. psyllaurous*, and two *Wolbachia* strains (Bin1 and Bin2) (Table 1). We provide detailed information on genome assemblies, annotations, and phylogenetic analyses in Supplemental Materials (Document S1; Table S1 to S6; Fig. S1 to S3).

**TABLE 1:** Metagenome assembly and annotation statistics for *B. cockerelli* symbionts

	*Carsonella-*BC-CA	*L. psyllaurous*	*Wolbachia*-Bin1	*Wolbachia*-Bin2
Total # reads	1,593,791	14,572,764	35,885,239	50,604,976
Average contig length	173,994	28,029	17,851	105,689
N50	173,994	91,145	34,417	347,941
Number of contigs	1	44	72	16
Genome size	173,994	1,233,258	1,285,302	1,690,987
Average depth of coverage	2025×	3,788×	6,303×	6,962×
Complete BUSCO (%)	100%	96%	55%	95%
Genes	231	1,176	1,543	1,722
CDS	201	1,133	1,513	1,684
tRNA genes	28	39	27	34
rRNA genes	2 (23S, 16S)	3	3	3
tmRNA	0	1	0	1

**Fig 2:**
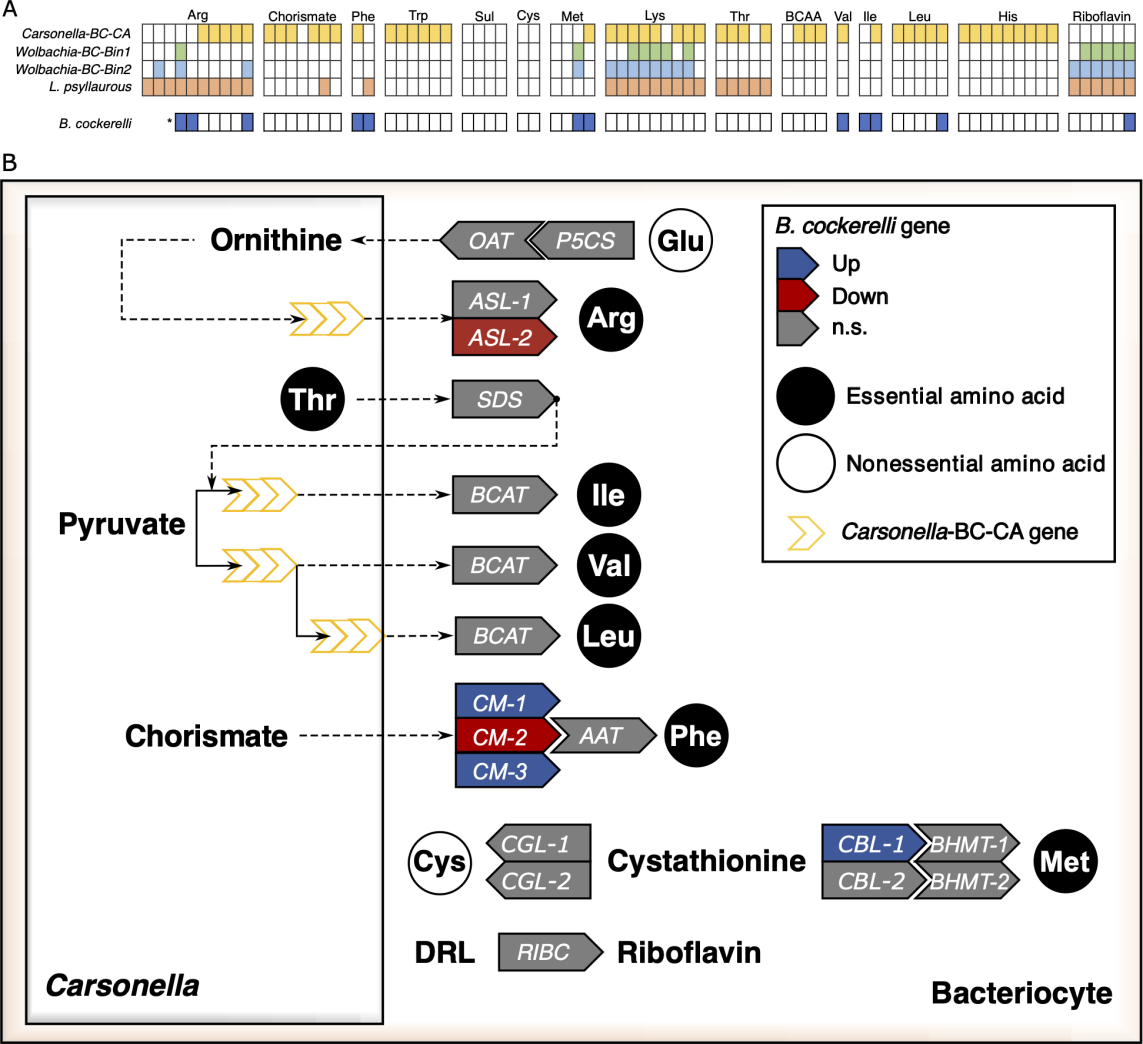
Metabolic reconstruction and differential gene expression involved in essential amino acid and riboflavin biosynthesis pathways in *B. cockerelli.* (**A**) Matrices of gene presence and absence in assembled *Carsonella*-BC-CA, *Wolbachia*-Bin1, -Bin2, *L. psyllaurous,* and *B. cockerelli* involved in essential amino acid and riboflavin biosynthesis pathways. Colored and white boxes represent the presence and absence of the gene, respectively. Collaborative *B. cockerelli* genes with *Carsonella* were identified in previous studies (32, 34). An asterisk indicates alternative, collaborative pathways in *B. cockerelli*. Arg, Arginine; His, histidine; Lys, lysine; Thr, threonine; BCAA, shared branched-chain amino acid pathway; Val, valine; Ile, isoleucine; Leu, leucine; chorismate, shared pathway for aromatic amino acids; Trp, tryptophan; Phe, phenylalanine; Met, methionine pathway (starting from the homoserine intermediate); Sul, sulfur reduction, and Cys, cysteine. (**B**) Differential gene expression patterns of *B. cockerelli* genes in *L. psyllaurous*-infected bacteriomes compared with uninfected bacteriomes. Genes that are significantly upregulated are shown in blue, and downregulated genes are shown in red, determined by a threshold of FDR *P*-value ≤ 0.05 and fold change (FC) ≥1.5×. Genes labeled “n.s.” indicate no significant change in gene expression.

Of the 10 essential amino acid biosynthesis pathways, *Carsonella*-BC-CA encodes complete pathways for histidine and tryptophan, near-complete pathways for phenylalanine, lysine, threonine, leucine, isoleucine, and valine, and incomplete or absent pathways for arginine and methionine, respectively (Fig. 2A; Table S7). The gaps in these pathways may be compensated by host-encoded genes and/or interactions with other symbionts (32–34, 38).

Interestingly, *L. psyllaurous* encodes complete pathways for arginine, lysine, and threonine biosynthesis, which are incomplete in *Carsonella* (Fig. 2A; Table S7). The *Wolbachia* strains, Bin1 and Bin2, retain incomplete pathways for all essential amino acids, further indicating their dependency on other symbionts like *Carsonella* or *L. psyllaurous* for nutrient provisioning (Fig. 2A; Table S7). However, both *L. psyllaurous* and *Wolbachia* strain Bin2 uniquely retain complete pathways for the essential B-vitamin riboflavin (Fig. 2A; Table S7), highlighting their potential roles in complementing host and symbiont nutrition.

### Effects of *L. psyllaurous* infection on insect host expression of symbiosis-related genes

Horizontally transmitted genes in the psyllid’s genome and other psyllid genes that collaborate with *Carsonella* for the intermediate and terminal steps of essential amino acid and riboflavin biosynthesis (Fig. 2A; Table S7) can serve as functional biomarkers for the enrichment of these *Carsonella* derived nutrients to the psyllid host. To understand how *L. psyllaurous* modulates the host’s collaborative biosynthesis of essential amino acids and vitamins with *Carsonella*, we compared gene expression patterns of *B. cockerelli* bacteriomes with and without *L. psyllaurous* infection.

From our RNA-seq experiment, an average of 21,522,796 (*N* = 6) and 20,839,516 (*N* = 6) high-quality reads per sample were successfully mapped to the *B. cockerelli* genome, covering approximately 67% and 68% of all trimmed reads in the *L. psyllaurous*-infected and uninfected samples, respectively (Table S8). Overall, we found that 11% of *B. cockerelli* genes were differentially expressed in bacteriomes due to *L. psyllaurous* infection, with 6% upregulated and 5% downregulated (Table S9). Most genes that collaborate with *Carsonella* for essential amino acid biosynthesis and riboflavin biosynthesis (*RIBC*) were not differentially regulated except for five genes, of which four were HTG (Fig. 2B; Table S10). Three of these genes collaborate with *Carsonella* for methionine and phenylalanine biosynthesis—specifically cystathionine beta-lyase (*CBL*-2) and two copies of chorismate mutase (*CM*-1 and *CM*-3)—were significantly upregulated, suggesting an increased output of *Carsonella* derived methionine and phenylalanine in the presence of *L. psyllaurous* (Fig. 2B; Table S10). However, the third chorismate mutase copy (*CM*-2) was downregulated, potentially indicating functional specialization among HGT copies in response to *L. psyllaurous* infection for phenylalanine biosynthesis. Notably, the HTG argininosuccinate lyase (*ASL*-2) was also significantly downregulated in *L. psyllaurous*-infected bacteriomes. This enzyme plays a crucial role in the final step of arginine biosynthesis (32, 33), suggesting a reduced output of arginine biosynthesis by *Carsonella* in the presence of *L. psyllaurous* (Fig. 2B; Table S10).

The biosynthesis pathways for the essential amino acids lysine and threonine, for which *Carsonella* encodes the majority of enzymes, do not have collaborative psyllid genes associated with them (Fig. 2A). Nevertheless, aspartate, which serves as the amino donor for *Carsonella*’s lysine and threonine biosynthesis pathways, is likely supplied by the psyllid host—similar to how *Acyrthosiphon pisum* provides aspartate to its obligate nutritional symbiont *Buchnera* (56). This is supported by the absence of asparaginase genes in *Carsonella*, *L. psyllaurous*, and the two *Wolbachia* strains (Table S7). *B. cockerelli’s* asparaginase gene was not differentially expressed in *L. psyllaurous*-infected compared with un-infected bacteriomes (Table S10). These results suggest that there is not an increased or decreased demand of host-derived aspartate in bacteriomes for *Carsonella* to produce lysine or threonine in the presence of *L. psyllaurous*.

### Modulation of *B. cockerelli* immune responses to *L. psyllaurous* in bacteriome and body tissues

Facultative symbionts often manipulate the host immune system to maintain colonization in different insect host cells and tissues without eliciting strong defense mechanisms, facilitating coexistence (57–60). Considering that *L. psyllaurous* is present throughout the psyllid’s lifecycle and found in both the bacteriome and body tissues (Fig. 1A) (49), we hypothesize that it may alter host immune pathways in both these cell types. To test this, we examined the gene expression patterns involved in the Toll and IMD pathways, given their critical role in insect immunity and symbiosis (61–64).

**Fig 3:**
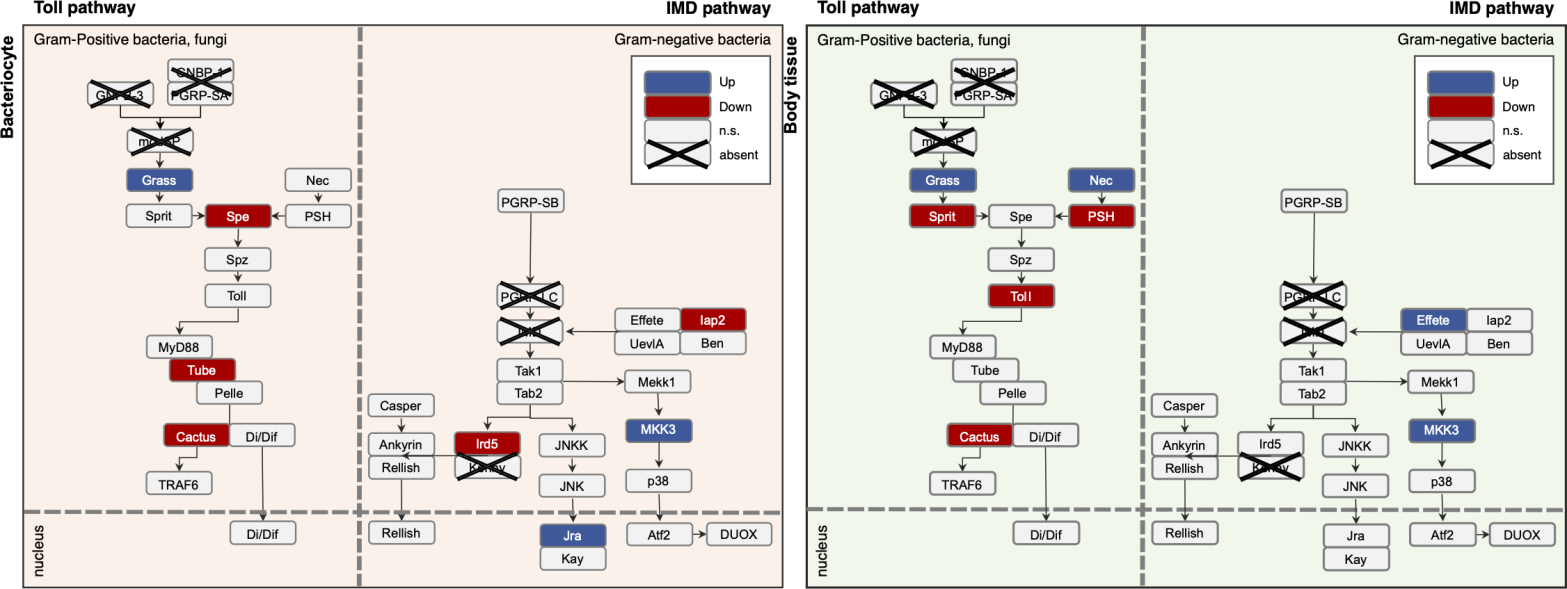
Schematic overview of differential gene expression patterns in the Toll and IMD pathways for *L. psyllaurous*-infected and uninfected *B. cockerelli* in bacteriomes and body tissues. Toll pathway (left panel) and IMD pathway (right panel) for both bacteriome and body tissues. *B. cockerelli* orthologs of immune genes were identified based on *D. citri* immune genes (66) and KEGG pathways (Table S11). Genes that are significantly upregulated are shown in blue, and downregulated genes are shown in red, determined by a threshold of FDR *P*-value ≤ 0.05 and fold change (FC) ≥1.5×. Genes absent in the *B. cockerelli* genome are indicated by an “X”. Pathways are organized by functional components, with the nucleus sectioning off downstream transcription factors based on KEGG pathways.

Toll receptor genes were significantly downregulated in *L. psyllaurous*-infected psyllids compared with uninfected ones for both tissue types down-stream of the activator, gram-positive specific serine protease (*GRASS*), which was upregulated (Fig. 3; Table S11). This indicates that the gram-negative symbiont *L. psyllaurous* may actively suppress its host’s Toll pathway, which generally responds to gram-positive bacteria and fungi in *Drosophila* (Fig. 3; Table S11) (65).

In contrast, the IMD pathway, typically responding to gram-negative bacteria (67, 68), showed a more nuanced regulatory response to *L. psyllaurous*. Key downstream components, such as mitogen-activation protein kinase 3 (*MAPKK3*) and the transcription factor *JRA*, were significantly upregulated in both infected bacteriomes and body tissues, indicating potential activation of parts of the IMD pathway. However, certain inhibitory genes, including Baculoviral IAP repeat-containing protein (*IAP2*) and I-kappaB kinase beta (*IRD5*), were down-regulated specifically in bacteriomes (Fig. 3; Table S11). In addition to the Toll and IMD pathways, several radical defense genes and clotting and melanization-associated genes were significantly upregulated in both bacteriomes and body tissues when *L. psyllaurous* is present. In contrast, several leucine-rich repeat containing proteins were downregulated in both bacteriomes and body tissues when *L. psyllaurous* was present (Table S11). These results indicate that a diversity of psyllid immune-related genes is significantly up- or down-regulated in the presence *L. psyllaurous*, with some genes responding in a tissue-specific manner (Fig. 3; Table S11).

### Transmission route of *L. psyllaurous* to psyllids does not require the egg pedicel

Vertical transmission plays a crucial role in the evolutionary stability and persistence of host-symbiont relationships (1). Although *Liberibacter* spp. can be horizontal transferred from plant to psyllid hosts through feeding, evidence of vertical transmission in *B. cockerelli* and *D. citri* via eggs has been documented in several studies using conventional PCR, qPCR, fluorescence *in situ* hybridization (FISH), transmission electron microscopy (TEM) and immunogold labeling (46, 69–72). However, the role of plants in facilitating this transmission to eggs remains unclear. Specifically, psyllid eggs are attached to host plant leaves by a pedicel, which is known to facilitate water uptake from the plant into psyllid eggs (73). In turn, this suggests another possible route for plant-mediated horizontal transmission.

To determine whether *L. psyllaurous* transmission to psyllid eggs depends on plants, we conducted oviposition experiments comparing *L. psyllaurous* presence and absence in eggs laid on plant-free, artificial diet oviposition sites with those laid on uninfected tomato plant leaves. We detected *L. psyllaurous* cell copies in both egg oviposition treatments (Fig. 1A), suggesting that *L. psyllaurous* does not require plant-mediated transmission via an egg pedicel to be transmitted into psyllid eggs. Interestingly, *L. psyllaurous* titers were significantly higher in eggs oviposited on plants compared with those laid on artificial diets (t = 2.8465, df = 5; Fig. 1A), with an average 8.8-fold increase.

### *L. psyllaurous* enhances *B. cockerelli*’s fitness

**Fig 4:**
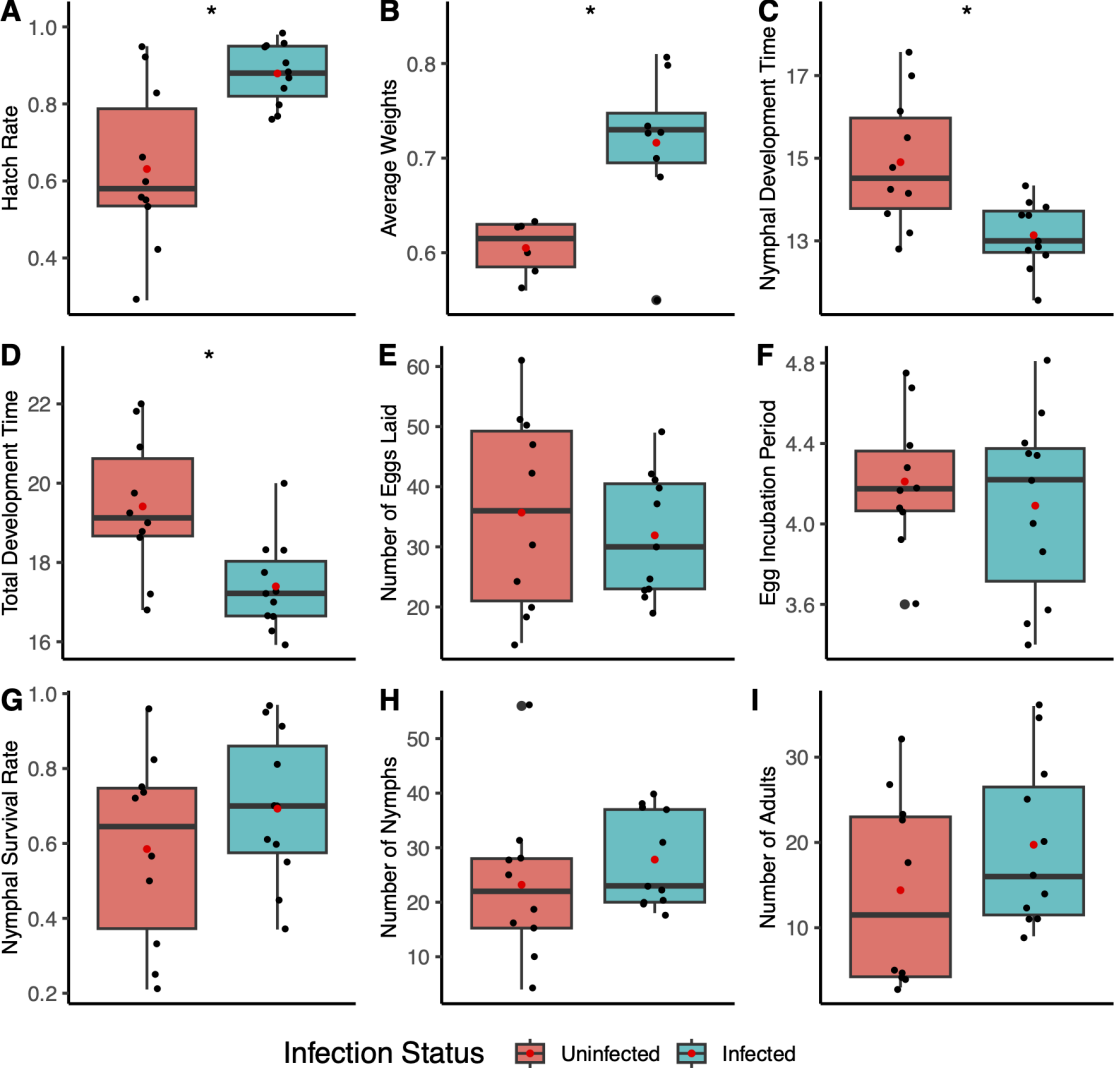
Comparison of life-history traits between *L. psyllaurous*-infected and -uninfected *B. cockerelli*. Box plots display the following fitness metrics per isofemale line: (**A**) hatch rate, (**B**) average weights, (**C**) nymphal development time, (**D**) total development time, (**E**) number of eggs laid, (**F**) egg incubation period, (**G**) nymphal survival rate, (**H**) number of nymphs, and (**I**) number of adults. Data points represent individual observations, with red dots indicating mean values. Statistical comparisons between *L. psyllaurous* infected and un-infected isofemale lines were performed using independent *t*-tests, with significance indicated by asterisks (*P < 0.05, **P < 0.01).

To assess the impact of *L. psyllaurous* on the fitness of *B. cockerelli*, we conducted bioassays on healthy, uninfected tomato plants measuring reproductive success, developmental rates, and survival with or without *L. psyllaurous* infection in the same genetic background as *B. cockerelli*. Our results indicate that *L. psyllaurous*-infected psyllids exhibited significantly greater fitness compared with uninfected psyllids, with a higher egg hatch rate (*P* = 0.003, F = 11.285; Fig. 4A) and average adult weights (*P* = 0.008, F = 10.273; Fig. 4B). Additionally, *L. psyllaurous* infected psyllids had significantly shorter development times in both their nymphal (*P* = 0.004, F = 10.611; Fig. 4C) and overall developmental stages (*P* = 0.005, F = 9.846; Fig. 4D). Other life-history traits were not significantly affected by *L. psyllaurous* infection (Fig. 4E through I). These results indicate that *L. psyllaurous* enhances developmental success, accelerating growth rates and increasing adult weights, all of which are associated with higher fitness in other insects (20, 21, 74).

### *L. psyllaurous* enhances *B. cockerelli* fitness under arginine-deprived conditions

Our metabolic reconstruction of *L. psyllaurous* indicates that *L. psyllaurous* has the metabolic potential to provide the essential amino acids lysine, threonine, and arginine, and the B-vitamin riboflavin to its psyllid host and resident microbes (Fig. 2A). This potential nutritional enhancement could help explain the improved fitness observed in *B. cockerelli* infected with *L. psyllaurous*. Our RNA-seq functional biomarker results however suggest that *L. psyllaurous*-infected psyllids have a reduced demand for only one of these essential nutrients—arginine—evidenced by the significant downregulation of the HTG *ASL* (Fig. 2B). To test whether *L. psyllaurous* helps its psyllid host compensate for arginine limitation, independent of host plant nutrition, we conducted bioassays comparing developmental time and adult weight of *L. psyllaurous*-infected compared with uninfected psyllids on artificial diets with and without arginine.

**Fig 5:**
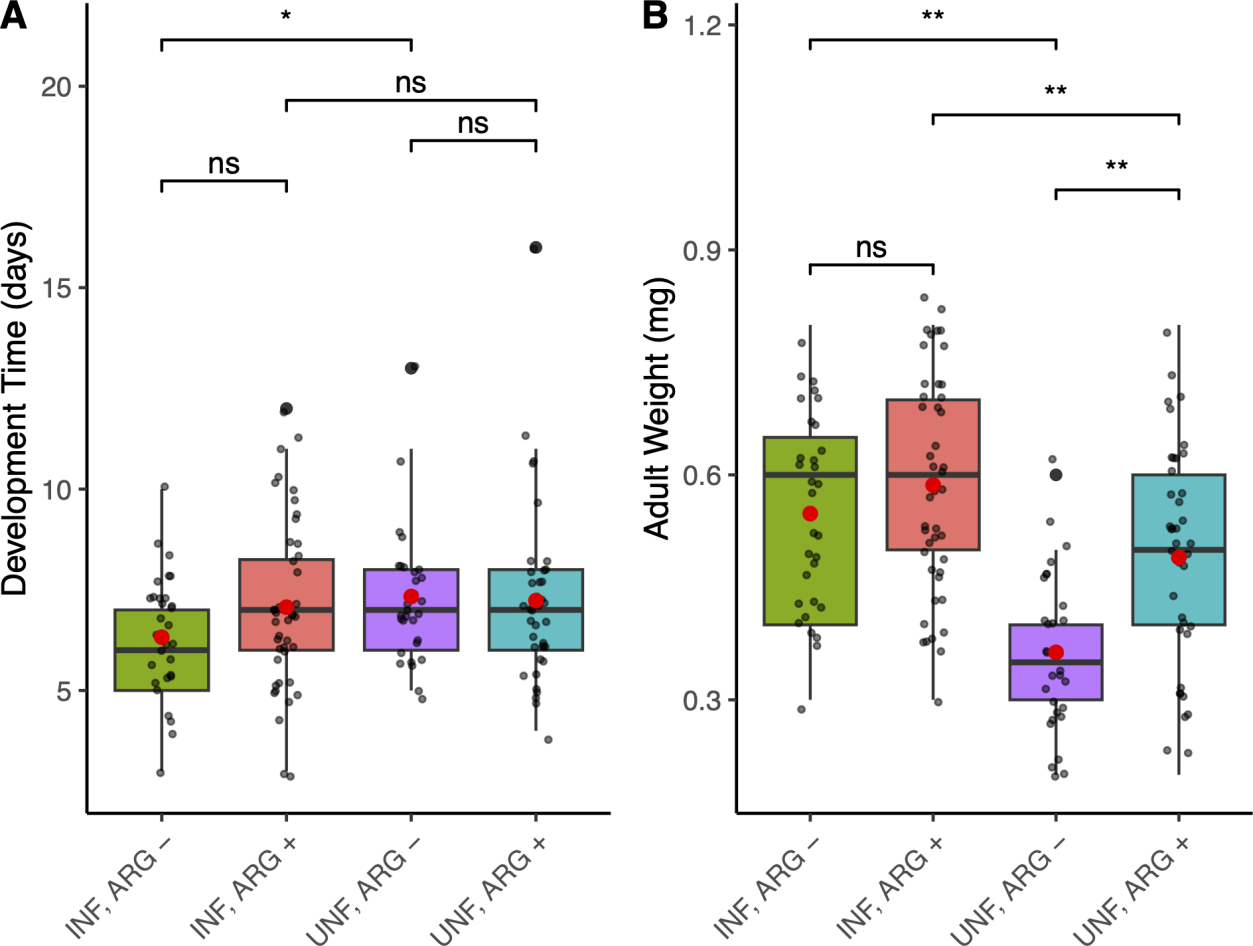
Enhanced fitness of *B. cockerelli by L. psyllaurous* when feeding on an arginine-deprived diet. (**A**) Development time (days) and (**B**) adult weight (mg) of *B. cockerelli* were measured across four treatments: *L. psyllaurous*-infected (INF) and -uninfected (UNF) psyllids reared on either an arginine-deprived (ARG-) or an arginine-supplemented (ARG+) artificial diet. Boxplots represent the median, interquartile range, and overall distribution of data points for each treatment group. Asterisks indicate significant pairwise differences (*P < 0.05, **P < 0.01), whereas “ns" denotes non-significant differences. Data points represent individual observations.

When comparing *L. psyllaurous* infected with uninfected psyllids, those feeding on an arginine-supplemented diet did not have significantly different developmental times (F = 0.118, df = 1.82, *P* = 0.732). However, when psyllids are feeding on an arginine-deficient diet *L. psyllaurous*-infected psyllids have significantly shorter developmental times compared with uninfected psyllids (F = 6.068, df = 1.60, *P* = 0.017) (Fig. 5A). Moreover, on both the arginine-supplemented diet (F = 9.049, df = 1.82, *P* = 0.004) and arginine-deficient diet (F = 37.243, df = 1.60, *P* < 0.001) *L. psyllaurous*-infected psyllids weigh significantly more than uninfected psyllids when feeding on the same diets; ~1.2× more on the arginine-supplemented diet and ~1.5× more on arginine-deficient diet (Fig. 5B). These results suggest that *L. psyllaurous* supports faster psyllid development when feeding on an arginine limited diet and increases psyllid body mass especially under arginine elimination.

## DISCUSSION

Facultative bacterial symbionts have previously been shown to confer benefits to insects; however, evidence of a specific nutritional benefit is rare for a symbiont that is not fixed in an insect population and obligate to an insect host (75), however, see Brownlie et al. (76). To our knowledge, our study provides the first evidence suggesting that a dual facultative symbiont and plant pathogen can benefit its insect host during amino acid limitation. Our manipulative artificial diet results corroborate our plant fitness, metagenomic, and transcriptomic results suggesting that *L. psyllaurous* provisions *B. cockerelli* with arginine, increasing insect fitness.

### Unique arginine pathway retention in *L. psyllaurous* highlights its nutritional role in *B. cockerelli*

This study also is the first report of a *Ca*. *Liberibacter* species playing a nutritional role in arginine provisioning for its host. Arginine is generally deficient or present in very low concentrations in plant phloem sap (77, 78). The retention of the entire arginine pathway in *L. psyllaurous* highlights its potential role in supplementing the insect host’s arginine requirements. Comparatively, other *Liberibacter* species, such as *L. asiaticus* and *L. africanus*, lack an enzyme required for converting glutamate to ornithine, a precursor in the arginine pathway (47, 79, 80). In contrast, *L. americanus* and *L. psyllaurous* retain this gene, encoding the entire arginine pathway (47, 81). As a result, *L. americanus* and *L. psyllaurous* may not rely on insect host-derived ornithine to synthesize arginine, as is hypothesized for their *Carsonella* symbiont (32). This suggests that unlike some other *Liberibacter* species, such as *L. asiaticus, L. psyllaurous* may have experienced selection pressure to retain the complete biosynthetic pathway for arginine biosynthesis, rather than rely on its host for the precursor metabolite, ornithine—particularly in environments where arginine is limited.

### *L. psyllaurous* increases *B. cockerelli* fitness

Our fitness results in this study were consistent for both our plant and artificial diet trials in showing that psyllids infected with *L. psyllaurous* have shorter developmental rates and higher adult masses compared with uninfected psyllids, especially during arginine elimination (Fig. 4 and 5). In contrast to our findings, two previous studies reported that *L. psyllaurous* could negatively impact the fitness of *B. cockerelli* on plants (82, 83). However, *L. psyllaurous*-infected and uninfected *B. cockerelli* lines in their studies were derived from two different genetic backgrounds (82, 83), suggesting that psyllid genotypes might have also contributed to the observed fitness differences between infected and uninfected psyllid lines. Our experimental design also differed from a prior study in having a longer premating period (5 days) (84), which aligns with ~4.2 ± 0.5 days needed for *B. cockerelli* to reach sexual maturity (85). Consequently, different psyllid and *Liberibacter* genotypes and experimental parameters may influence fitness results. Future trials, especially under field conditions, are necessary to understand *L. psyllaurous’s* influence more fully on *B. cockerelli* fitness under fluctuating environmental conditions. For example, in aphids, the facultative symbiont *Hamiltonella defensa* is mutualistic and confers defense to parasitoid wasps; however, depending on environmental context (e.g., absence of parasitoid) and *H. defensa* strain types, this symbiont can also have negative fitness effects on the aphid host (86–89).

*Candidatus L. asiaticus* also increases psyllid host fitness (22–24). A recent study revealed that the increased fecundity in *D. citri* has been linked to the modulation of hormonal pathways, particularly adipokinetic hormone (AKH) signaling (90). In our RNA-seq data, we found that the AKH receptor (*AKHR*) gene was not differentially expressed in either *L. psyllaurous*-infected bacteriome or body tissues compared with uninfected tissues (Table S11). The *AKH* gene is present in the *B. cockerelli* genome; however, the expression of this gene was not detected in our analysis (Tables S9 and 11). Similarly, in another study on the gene expression patterns of *B. cockerelli* under *L. psyllaurous* infection (91), the *AKH* and *AKHR* genes were not differentially regulated in *L. psyllaurous*-infected ovaries compared with salivary glands. Collectively, these results suggest that the mechanisms by which *L. psyllaurous* enhances fitness in *B. cockerelli* may differ from the hormonal modulation observed in *L. asiaticus*-infected *D. citri*.

### *L. psyllaurous* does not require the egg pedicel for transmission to egg

Symbionts that are both vertically and horizontally transmitted to insect hosts such as *L. psyllaurous* are generally not expected to evolve into obligate, fixed mutualistic relationships (92). Instead, host populations can potentially maintain strain diversity, which has been observed in other mutualistic host-symbiont systems, especially in fluctuating environments (93). Currently, it is unclear if *L. psyllaurous* can also be transmitted through the egg pedicel, which could potentially serve as an additional route of horizontal transmission alongside feeding. Here, our results demonstrate that although *L. psyllaurous* transmission via eggs does not depend on the plant-mediated mechanisms via egg pedicel, *L. psyllaurous* genome copy numbers were higher when psyllids oviposit on un-infected plant leaves versus sterile artificial diet (Fig. 1A). This result suggests that either additional transfer occurs through the egg pedicel from the plant environment or that the plant nutrient environment enhances the proliferation of maternally transmitted *L. psyllaurous* in psyllid eggs. For example, in *Bemisia tabaci*, nutrient transfer from the plant to the egg has been demonstrated previously through the pedicle (94).

### Interactions of *L. psyllaurous* with its resident microbiota and insect host immunity

The abundance of genome copies of *L. psyllaurous* and *Wolbachia* in bacteriomes (Fig. 1) suggests that these symbionts may benefit from this nutrient-rich environment by receiving some essential amino acids from *Carsonella*. Given that *L. psyllaurous* does not significantly influence the genome copy numbers of *Wolbachia*-Bin1 or Bin2, there appears to be no strong competitive interactions between *L. psyllaurous* and the *Wolbachia* strains in bacteriomes or body tissues (Fig. 1). Instead, potentially *L. psyllaurous* benefits from the presence of *Wolbachia* in *B. cockerelli*. For example*, Wolbachia*-Bin1 encodes a repressor protein homolog (Document S1) that was previously identified in *Wolbachia* from *D. citri* to repress the lytic phage gene holin in *L. asiaticus* (54). This repressor protein may also play a role in repressing phage activity of *L. psyllaurous* when this symbiont is inside of its psyllid host environment. Such modulation could be instrumental in helping both *Wolbachia* and *L. psyllaurous* evade host immune responses, thereby promoting their survival and enhancing their transmission rates. This finding may explain why *Wolbachia*-infected biotypes of *B. cockerelli* exhibit higher acquisition and transmission rates of *L. psyllaurous* compared with the *Wolbachia*-free Northwestern biotype (53).

Like other facultative symbionts of insects, *L. psyllaurous* occurs not just in the bacteriome but in other insect host tissues as well (49, 95–97). In contrast to the reference genome of *L. psyllaurous* (47), our genome assembly of *L. psyllaurous* encodes an intact secB gene, suggesting that the Sec pathway may be functional in certain *L. psyllaurous* strains, including the haplotype B strain assembled here (Document S1). A previous transcriptomic analysis reported active expression of *secB* within *B. cockerelli*, supporting its functional relevance in some *L. psyllaurous strains* (98). The presence of an operational Sec pathway, alongside the type I secretion system (T1SS) and its associated toxins (e.g., HlyD, PrtD, and RTX) (47), indicates that *L. psyllaurous* may use these systems to modulate host immunity and establish infection in one or both of its hosts.

Our RNA-seq results from both bacteriome and body tissues reveal that *L. psyllaurous* infection can suppress *B. cockerelli’s* Toll pathway and modify other immune-related genes including the IMD pathway, potentially allowing the bacterium to evade immune detection to maintain itself inside of its psyllid host (Fig. 3; Table S11). The IMD pathway in *B. cockerelli* appears reduced compared with insects with complete metamorphosis (Fig. 3; Table S11), a trend observed previously in other hemipterans such as pea aphids (99), *D. citri* (66), and the brown-winged green stinkbug, *Plautia stali* (63). Interestingly, in *P. stali*, the Toll and IMD pathways demonstrate functional crosstalk, with the Toll pathway being activated by both gram-positive and gram-negative bacteria (63). This indicates that in certain hemipteran insects, the regulation of these pathways in response to gram-negative bacteria—many of which are symbionts—may differ dramatically from the immune responses seen in other insect models, such as *Drosophila*.

In conclusion, we found evidence that a dual facultative symbiont, *L. psyllaurous,* can modify the immune pathways of its insect host and provide benefits when arginine is limited. This discovery highlights the diverse ecological and evolutionary roles of facultative symbionts to their insect host in improving insect fitness. Future studies exploring various psyllid biotypes and *L. psyllaurous* haplotypes under fluctuating arginine diet conditions will provide deeper insights into the dynamics of this unique host-symbiont nutritional relationship.

## MATERIALS AND METHODS

### Experimental psyllid lines

Experimental psyllid lines (*L. psyllaurous*, uninfected and infected) were established using the same genetic background as *B. cockerelli*. The uninfected *B. cockerelli* line was derived from a wild population in Temecula, California, USA, in August 2019 (CA), which has the genetic background used for the sequencing and chromosomal assembly of the *B. cockerelli* genome (isoF-IL) (34). The *L. psyllaurous* strain used in this study originated from an infected wild population from Weslaco, Texas, USA, collected in July 2017 (TX-Lpsy).

To generate the infected *B. cockerelli* line with the same genetic background as the uninfected line (CA), 15 infected male *B. cockerelli* from the TX-Lpsy line were introduced onto 4- to 6-week-old tomato plants (*S. lycopersicum* var. Moneymaker) and were allowed to feed for 2 weeks. Subsequently, all psyllids were removed from the tomato plants, and the top-tier leaves were screened for *L. psyllaurous* infection using real-time quantitative PCR (qPCR) (detailed in (Document S3)). Once *L. psyllaurous* infection was confirmed, 10–15 uninfected *B. cockerelli* (both sexes) from the CA line were transferred onto the infected tomato plants to generate the CA-Lpsy line and were maintained on 8- to 12-week-old tomato plants (*Solanum lycopersicum* var. Moneymaker) at 25°C under a 16L:8D light/dark cycle for several generations before trials.

### Symbiont quantification

The normalized ratio value (NV) for *L. psyllaurous* and *Wolbachia* genome copies relative to psyllid genome copies was calculated for three biological replicates using the following equation: average symbiont DNA single gene copy quantity/average psyllid DNA single gene copy quantity based on the standard curve protocol outlined in Bookout et al. (100). For *L. psyllaurous*, statistical analysis of the NV was conducted using an independent samples *t*-test to determine significant (*P* < 0.05) differences between tissue types. For *Wolbachia*, statistical analysis of NV was conducted between two factors (infection status and tissue type) using a two-way ANOVA. All statistical analyses were conducted in R version 4.4.0 and visualized with box plots using the ggplot2 package (101).

### Metagenomic sequencing and symbiont genome assembly

Symbiont cells of *L. psyllaurous* were filtered from 0.3 g of mixed age infected *B. cockerelli* from both sexes. Filtration followed the protocol described in Hansen and Moran (2012) (102) with modifications. Illumina library preparation and sequencing for extracted symbiont DNA were performed at the University of California, Riverside Genomics Core facility. Further details on symbiont cell extraction and metagenomic sequencing, assembly, and phylogenetic analyses are provided in Document S3 and Table S12.

### Annotation and analysis of symbiont genes in the amino acid biosynthetic metabolism

To determine the metabolic capabilities of assembled symbionts, we annotated genes involved in the amino acid metabolism similar to Kwak et al. (2023) (34). Briefly, BlastKOALA (103) was used to assign KO (K number) for each symbiont, including protein sequences from the reference genomes. Amino acid biosynthetic pathways were then analyzed using KEGG Mapper, Reconstruct (104). To ensure accuracy, NCBI-BLAST with an E value cutoff of 10e-10 was further used for manual curation to identify any missing genes compared with the reference symbiont genomes.

### RNA-seq and differential gene expression analysis

RNA-seq was performed on dissected bacteriome and body tissues from fifth instar nymphs of both *L. psyllaurous*-infected (CA-Lpsy) and uninfected (CA) *B. cockerelli* lines. Total RNA was extracted, and strand-specific libraries were prepared and sequenced on the Illumina NovaSeq S4 platform. Differentially expressed genes were identified by comparing infected and uninfected tissues using EdgeR (105), with significance defined by FDR ≤ 0.05- and ≥1.5-fold change in normalized expression. Additional details on tissue dissection, RNA extraction, and bioinformatics pipelines are provided in Document S3.

### *B. cockerelli* immune gene identification and annotation

To annotate immune-related genes in the *B. cockerelli* genome (34), *D. citri* immune proteins were obtained from Arp et al. (66), and KEGG pathways for the Toll and IMD signaling pathways for *D. citri*. For genes missing from *D. citri* within the Toll and IMD signaling pathways, protein sequences were obtained from either KEGG pathways for *A. pisum* or *Drosophila melanogaster* if present. OrthoVenn3 (106) using the OrthoMCL algorithm was then used to determine if *B cockerelli* possessed orthologs with any of the immune-related proteins annotated from the latter insect taxa.

### Assessment of *L. psyllaurous* vertical transmission

To assess the vertical transmission of *L. psyllaurous* through eggs, we conducted oviposition assays on both uninfected tomato plants and sterile artificial diet arenas. For plant oviposition assays, 40 1-week-old *L. psyllaurous*-infected male and female psyllids were collected and allowed to feed and mate on a 4-week-old uninfected tomato plant for 24 h. After 24 h, all psyllid adults were removed, and eggs were subsequently collected using sterile insect pins. In artificial diet oviposition assays, we collected 10 1-week-old *L. psyllaurous*-infected male and female psyllids. These psyllids were then allowed to feed and mate for 24 h inside of a petri dish arena (*n* = 3). We employed the same artificial diet and Petri dish arena design as in Pers and Hansen (107). In brief, the Petri dish arena consists of a bottom dish filled with the diet. This diet is shielded from psyllids by two layers of stretched parafilm topped with a mesh cover. Although psyllid mouthparts can penetrate through both layers of parafilm to feed, it is noteworthy that egg pedicels were exclusively laid on the outer layer of the parafilm and did not breach this barrier. After adults were removed 24 h after introduction into the arena the top layer of parafilm was removed and placed on a new sterile Petri dish. Like plant treatments, eggs were collected using sterile insect pins 24 h after psyllid adults were removed.

For both the plant and diet, treatments eggs were bleach treated and triple rinsed similar to Casteel et al. (46). For DNA extractions, we utilized the Quick-DNA Microprep Plus Kit, pooling five eggs per biological replicate with 3–4 biological replicates per treatment. The titer of *L. psyllaurous* in the eggs was determined using qPCR (see Symbiont quantification above; Document S3). The lower end of the standard curve, uninfected egg controls, and non-template-negative controls were used to establish the limit of *L. psyllaurous* detection.

### Fitness trial

Ten isofemale lines were established to ensure uniform progeny cohorts for fitness trials, detailed in Document S3. The following life history and developmental data were then collected for each isofemale line: total eggs oviposited (the number of eggs laid), total nymphs emerged, total adults, hatching rate (the percentage of eggs that hatched), incubation time (the average period from the first egg appearance to the first nymph emergence), nymphal survival rate (the percentage of nymphs that survived to adulthood), nymphal development time (the average time period from the first nymph appearance to the first adult emergence), and total development time (the average time from the first egg appearance to the first adult emergence). Diagnostic qPCR for *L. psyllaurous* (Document S3) was conducted before and after trials to confirm infected psyllids were infected and uninfected psyllids were uninfected before and after trials. Statistical analysis was performed on the life history and developmental data collected during the fitness trials to determine the effects of *L. psyllaurous* infection on *B. cockerelli*. The Levene’s Test of Equality of variance was conducted to ensure all variables had equal variances followed by an Independent Samples *t*-test or a two-factor ANOVA using IBM SPSS statistics v29.0.2.0 (108).

### Diet manipulation of arginine with *L. psyllaurous-*infected and -uninfected psyllids

Diet manipulation experiments were conducted on *L. psyllaurous*-infected and -uninfected *B. cockerelli* to assess the impact of arginine deprivation. Third instar nymphs were cultured on uninfected tomato plants before being transferred to one of four artificial diet treatments: (i) (Control Diet with Arginine, Not Infected): Psyllids not infected with *L. psyllaurous*, fed with a control diet containing all amino acids, vitamins, salts/buffers/sterols, and trace metals as in Pers and Hansen (107); (ii) (Control Diet with Arginine, Infected): Similar to Group 1 except psyllids were infected with *L. psyllaurous*; (iii) (Arginine-Deprived Diet, Not Infected): Psyllids not infected with *L. psyllaurous*, fed a similar diet as controls except with a diet lacking arginine but compensated with glutamate to maintain the same nitrogen content as the control diet, similar to Vogel and Moran (109); and (iv) (Arginine-Deprived Diet, Infected): Similar to Group 3 except psyllids infected with *L. psyllaurous*. Feeding chambers were set up to facilitate diet delivery. Nymphal development time and adult weights were recorded. Further details on diet composition, feeding chamber design, and bioassay statistics are provided in Table S13 and Document S3.

## Data Availability

Raw RNA-seq reads, symbiont metagenomic reads, symbiont 16S rRNA sequences, and symbiont genome assemblies have been deposited at NCBI under BioProject accession number PRJNA1082796. All code used in this paper is from the “Dataset_S9_RNAseq_Code” (110), which is publicly available on figshare: https://doi.org/10.25387/g3.14109851.

## SUPPLEMENTAL MATERIAL

Document S1 mbio.03588-24-s0001.pdf Supplemental results on endosymbiont genome assemblies, annotations, and phylogenetic analyses.

Document S2 mbio.03588-24-s0002.pdf Tables S1-S5, S8, S10, S12, and S13 and Figures S1-S3.

Document S3 mbio.03588-24-s0003.pdf Supplemental methods.

Table S6 mbio.03588-24-s0004.xlsx Best BLASTx hits to the nr database for amino acid sequences of the *Wolbachia*-Bin1 and -Bin2 assembly singletons identified in Orthovenn3.

Table S7 mbio.03588-24-s0005.xlsx The repertoire of *Carsonella*-BC-CA, *L. psyllaurous*, and *Wolbachia*-Bin1 and -Bin2 genes involved in amino acid biosynthesis pathway.

Table S9 mbio.03588-24-s0006.xlsx RNA-seq raw expression and differential expression analysis data for tissue comparisons in response to *L. psyllaurous* infection.

Table S11 mbio.03588-24-s0007.xlsx Gene expression data for immune and hormone modulation.

ASM does not own the copyrights to Supplemental Material that may be linked to, or accessed through, an article. The authors have granted ASM a non-exclusive, world-wide license to publish the Supplemental Material files. Please contact the corresponding author directly for reuse.
