# Supplementary material for: A dual insect symbiont and plant pathogen improves insect host fitness under arginine limitation
Source: mBio. 2025 Feb 25;16(4):e03588-24. doi: 10.1128/mbio.03588-24 (PMC11980576; doi:10.1128/mbio.03588-24)
Supplement: Document S1 — Supplemental results on endosymbiont genome assemblies, annotations, and phylogenetic analyses. [file mbio.03588-24-s0001.pdf]

Metagenomic assembly and annotation of *B. cockerelli* symbionts

Carsonella de novo assembly

Metagenomic sequencing on filtered bacterial cells of *L. psyllaurous*-infected *B. cockerelli* yielded 362,410,457 high-quality reads from a total of 414,028,150 raw reads (**Table S1**). *Carsonella* reads were de novo assembled into a single contig/chromosome with ~2,025X coverage, hereafter named *Ca.* Carsonella ruddii - *B. cockerelli* California strain (*Carsonella*-BC-CA) (**Table 1**). Comparison with the reference *Carsonella*-BC (NZ_CP019943.1; 1) from the same host species showed high similarity, with 98.26% ANI and genome lengths of 173,994 bp and 173,802 bp, respectively. Both assemblies exhibited identical %GC content (14.8%), gene order, and BUSCO scores, emphasizing the consistency between genomes (**Table S2**). Prokka and BLASTp analyses confirmed the same gene number and identify between both genomes (**Table S3**).

Strain differences were expected due to geographical origins: *Carsonella*-BC-CA from California, USA, and *Carsonella*-BC-WA from Washington, USA (hereafter referred as *Carsonella*-BC-WA ; reference 1). The 16S rRNA gene sequences were compared with other *B. cockerelli Carsonella* sequences from the Western (AF211126.2) and the Northwestern biotypes (KR045612.1) of *B. cockerelli* (2). *Carsonella*-BC-CA and *Carsonella*-BC-WA had 99.6% similarity in the 16S rRNA gene (1512/1518 nucleotide identity; **Table S4**). *Carsonella*-BC-CA was more similar to the Western haplotype (99.9 % identity), while *Carsonella*-BC-WA showed 99.6 % similarity, consistent with collection locations for the two different psyllid biotypes (**Table S4**; 2).

L. psyllaurous de novo assembly

For the draft genome assembly of *L. psyllaurous,* 44 contigs were *de novo* assembled with an average depth of coverage of ~3,788X ± 310 (SE) and an N50 of 91,145 (**Table 1**). Of the 44 contigs, the majority (34) were largely syntenic in comparison to the reference genome (NC_014774.1; 3). The de novo assembly showed high similarity to the reference genome (3), with an ANI of ~99.57%, a genome length of 1,233,258 bp vs 1,258,278 bp, %GC was 35.1 vs 35.2, and BUSCO scores of 500 vs 492 for the "complete single copy" category (**Tables S2**). The reference assembly was missing six more BUSCOs, identified as pseudogenes due to frameshift mutations (*rpsG*, *holA*, *nuoJ, secB*, *yggT*, and *rsfS).* No multicopy BUSCOs were found in either the reference or *de novo* assembly.

After final annotation and curation, the *de novo* assembly shared 1,040 genes with the reference genome (3), excluding orthologous gene copies based on OrthoVenn3. In total there are 30 more CDS observed in the reference genome compared to the *de novo* assembly, mainly multicopy, with nine unique CDS comprising eight hypothetical genes and one annotated gene (3-methyladenine DNA glycosylase). All nine coding sequences in the reference matched *Liberibacter* spp. Six unique CDS in the de novo assembly were hypothetical genes, five matching to *Liberibacter* spp. and one CDS had a best BLAST hit to a *L. psyllaurous* phage CDS (**Table S5**).

To determine the *L. psyllaurous* haplotype, the 16S rRNA gene from the *de novo* assembly was compared to known haplotypes listed in Cooper et al. (2022) (4) and showed 100% nucleotide similarity to *Ca*. Liberibacter psyllaurous haplotype B (FJ829813.1; 5).

Wolbachia de novo assemblies

PacBio and Illumina reads (see methods) were used to assemble two *Wolbachia* strains*.* Based on Anvio v8.0 (6) binning results, two different strains (referred to hereafter as *Wolbachia*-Bin1 and -Bin2) were identified. These strains were distinguished by divergent copies of the *wsp* gene, which is rapidly mutating and used for strain identification (7, 8).

Previously, two strains of *Wolbachia* were found to co-occur in field collected *B. cockerelli* individuals based on unique *wsp* sequences, strains A and B (8, 9). The *wsp* gene in *Wolbachia*-Bin1

showed 100% nucleotide similarity to strain A (KM267306.1), while the wsp gene in *Wolbachia*-Bin2 matched strain B (KM267307.1) with 100 % similarity. Pairwise comparison showed 86.26% similarity between the *wsp* genes of the two strains. In turn, for subsequent analyses here *Wolbachia* contigs in Bin1 and Bin2 were examined as two separate *de novo* draft assemblies. Nevertheless gene content comparisons between Bins1 and 2 should be approached cautiously due to the potential collapse of highly similar genes into one bin or the other given strains cannot be isolated separately because of their co-infection status in individual psyllids (8, 9).

Wolbachia Bin1 assembled into 72 contigs with an average depth of coverage of 6,303x ± 389 (SE), an N50 of 34,417bp, and a genome length of 1,285,302bp (**Table 1**). *Wolbachia* Bin2 assembled into 16 contigs with an average depth of coverage of ~6,962x ± 350 (SE), an N50 of 347,941bp, and a genome length of 1,690,987bp (**Table 1**). We compared our two *Wolbachia* assemblies (Bins1 and 2) to a *Wolbachia* assembly from *D. citri* (referred to hereafter as *Wolbachia*-DC)(NZ_CP048820.1; 10). The percent GC of both genome bins showed ~ 34%, similar to the genome of *Wolbachia*-DC. To determine genome completeness, *Wolbachia*-Bin1 had 201 complete BUSCOs of 364, while Bin2 had 346 complete BUSCOs, which is comparable to the *Wolbachia*-DC genome assembly with 344 complete BUSCOs (**Table S2**). The number of annotated CDS for *Wolbachia*-Bin1 was 1,513, which was lower than *Wolbachia*-Bin2, which had 1,684 annotated CDS. Among them, *Wolbachia*-DC had the lowest CDS count, with 1,454 annotated CDS (**Table S3**).

Using OrthoVenn3 (11), a total of 595 orthologous clusters containing 2,125 proteins (436 that are single copy) were shared among all three *Wolbachia* assemblies (**Figure S1**). A total of 151 orthologous clusters (357 proteins) were exclusively shared between *Wolbachia*-Bins1 and 2, while 353 orthologous clusters (758 proteins) were specifically shared between *Wolbachia*-Bin2 and *Wolbachia*-DC, and 48 orthologous clusters (138 proteins) were uniquely shared between *Wolbachia*-Bin1 and *Wolbachia*-DC (**Figure S1**). GO terms were significantly enriched for shared orthologs for translation GO:0006412 ($p<3.82e-09$) and the biosynthetic process GO:0009058 ($p<0.0032$) in *Wolbachia*-Bin1 and *Wolbachia*-DC

Gene clusters unique to each *Wolbachia* assembly consist of 59 clusters (202 proteins) for Bin1, 42 clusters (118 proteins) for Bin2, and 27 clusters (68 proteins) for *Wolbachia*-DC (**Figure S1**). Significantly enriched GO terms for unique orthologs in Bin1 were transposition GO:0032196 ($p<0.00055$) and response to X-ray GO:0010165 ($p<0.00257$). Only one GO term, transposition GO:0032196, was significantly enriched ($p<1.02e-07$) for *Wolbachia*-DC unique orthologs.

Each of the *Wolbachia* assemblies, *Wolbachia*-Bin1, *Wolbachia*-Bin2, and *Wolbachia*-DC, possess unique singleton genes, with 245 singletons in *Wolbachia*-Bin1, 260 in *Wolbachia*-Bin2, and 227 in *Wolbachia*-DC (**Table S6**). A total of 45% singletons in *Wolbachia*-Bin1, 42% singletons in *Wolbachia*-Bin2, and ~96% singletons in *Wolbachia*-DC had significant matches to the NCBI nr database and were primarily composed of hypothetical genes from other *Wolbachia* species. Singletons from both Bin1 and Bin2 that had best BLAST hits to annotated genes are primarily composed of genes associated with protein-protein interaction (e.g., ankyrin repeat domain-containing proteins), DNA replication and repair, and phage-associated protein (see **Table S6** for more detail).

To identify putative *Wolbachia*-BC proteins related to the psyllid's vector competency, we conducted a BLASTp analysis against our *Wolbachia*-Bin1 and Bin2 assembly using a 56 amino acid protein of *Wolbachia*-DC (NCBI accession number WP_017531870.1), which was previously identified to be involved in suppressing phage lytic cycle genes of *L. asiaticus*. We identified a protein homolog of this repressor protein, WP_017531870.1, from *Wolbachia*-Bin1 with 65% amino acid identity with e-value of 1.87e-14.

Phylogenetic analyses of *Wolbachia* strains from Bin1 and Bin2

Analyses of the *wsp*, 16S rRNA, and core orthologous genes with Cafe5 indicate that the *Wolbachia* Bin1 and Bin2 co-infecting *B. cockerelli* are distinct strains that diverged ~1 million years ago (**Figure S2 and S3**). BLAST similarity and pairwise DNA--DNA hybridization (DDH) analyses further

support that these genomes represent different *Wolbachia* species that diverged in the distant past. The ANI between Bin1 and Bin2 assemblies is ~94.1% and the DDH estimate was ~61.3%, with a confidence interval of 58.4 – 64%, which is below the empirical thresholds typically used for assigning genomes to the same subspecies (79%) or even species (70%) (12).

Phylogenetic analyses of single-copy orthologs and 16S rRNA situate Bin1 and Bin2 as a discrete clade within the supergroup B of *Wolbachia* (**Figure S2**). *Wolbachia* Bin1 and Bin2 are most closely related to *Wolbachia* harbored in the brown planthopper (*Nilaparvata lugens*) rather than those in *D. citri* or the whitefly (*Bemisia tabaci*), indicating horizontal transmission among these insect species (**Figure S2**). The *wsp* gene phylogeny was discordant with the single copy ortholog and the 16S rRNA phylogenetic trees, indicating that recombination of the *wsp* gene has occurred among strains similar to previous observations in *Wolbachia* (13).

The 16S rRNA phylogenetic analyses demonstrate that *Wolbachia* Bin1 and Bin2 are identical to the two strains previously detected in the Western and Central biotypes of *B. cockerelli* (8, 9). Interestingly, the most divergent *B. cockerelli* haplotype (Northwestern) is known to possess only one *Wolbachia* strain related to Bin1 based on 16S rRNA amplicon sequencing (**Figure S2**). While the Western and Central haplotypes harbor two strains, the Northwestern haplotype generally does not harbor *Wolbachia*, as confirmed via PCR (4).

Gene expansion and contraction analyses show that the lineage leading to *Wolbachia* Bin1 and Bin2 displays an expansion of five gene clusters (75 proteins) that are significantly enriched ($P<0.05$) for the following GO terms: serine-type peptidase activity, metallopeptidase activity, transposition, sequence-specific DNA binding, transcription, and DNA-templated (**Figure S3**). Conversely, three contracting gene clusters (eight proteins) leading to the *Wolbachia* Bin1 and Bin2 lineage are significantly enriched for the following GO terms: protein-cysteine S-palmitoyltransferase activity, defense response, and exocytosis (**Figure S3**).

Reference

1. Riley AB, Kim D, Hansen AK. 2017. Genome Sequence of "*Candidatus* Carsonella ruddii" Strain BC, a Nutritional Endosymbiont of *Bactericera cockerelli*. Genome Announcements 5:10.1128/genomea.00236-17.
2. Fu Z, Meier AR, Epstein B, Bergland AO, Castillo Carrillo CI, Cooper WR, Cruzado RK, Horton DR, Jensen AS, Kelley JL, Rashed A, Reitz SR, Rondon SI, Thinakaran J, Wenninger EJ, Wohleb CH, Crowder DW, Snyder WE. 2020. Host plants and *Wolbachia* shape the population genetics of sympatric herbivore populations. Evolutionary Applications 13:2740–2753.
3. Lin H, Lou B, Glynn JM, Doddapaneni H, Civerolo EL, Chen C, Duan Y, Zhou L, Vahling CM. 2011. The Complete Genome Sequence of '*Candidatus* Liberibacter solanacearum', the Bacterium Associated with Potato Zebra Chip Disease. PLOS ONE 6:e19135.
4. Cooper WR, Horton DR, Swisher-Grimm K, Krey K, Wildung MR. 2022. Bacterial Endosymbionts of *Bactericera maculipennis* and Three Mitochondrial Haplotypes of *B. cockerelli* (Hemiptera: Psylloidea: Triozidae). Environmental Entomology 51:94–107.
5. Wen A, Mallik I, Alvarado VY, Pasche JS, Wang X, Li W, Levy L, Lin H, Scholthof HB, Mirkov TE, Rush CM, Gudmestad NC. 2009. Detection, Distribution, and Genetic Variability of '*Candidatus* Liberibacter' Species Associated with Zebra Complex Disease of Potato in North America. Plant Disease 93:1102–1115.
6. Eren AM, Kiefl E, Shaiber A, Veseli I, Miller SE, Schechter MS, Fink I, Pan JN, Yousef M, Fogarty EC, Trigodet F, Watson AR, Esen ÖC, Moore RM, Clayssen Q, Lee MD, Kivenson V, Graham ED, Merrill BD, Karkman A, Blankenberg D, Eppley JM, Sjödin A, Scott JJ, Vázquez-Campos X, McKay LJ, McDaniel EA, Stevens SLR, Anderson RE, Fuessel J, Fernandez-Guerra A, Maignien L, Delmont TO, Willis AD. 2021. Community-led, integrated, reproducible multi-omics with anvi'o. Nat Microbiol 6:3–6.
7. Zhou W, Rousset F, O'Neill S. 1998. Phylogeny and PCR-Based Classification of *Wolbachia* Strains Using WSP Gene Sequences. Proceedings: Biological Sciences 265:509–515.
8. Cooper WR, Swisher KD, Garczynski SF, Mustafa T, Munyaneza JE, Horton DR. 2015. *Wolbachia* Infection Differs Among Divergent Mitochondrial Haplotypes of *Bactericera cockerelli* (Hemiptera: Triozidae). Annals of the Entomological Society of America 108:137–145.
9. Liu D, Trumble JT, Stouthamer R. 2006. Genetic differentiation between eastern populations and recent introductions of potato psyllid (*Bactericera cockerelli*) into western North America. Entomologia Experimentalis et Applicata 118:177–183.
10. Neupane S, Bonilla SI, Manalo AM, Pelz-Stelinski KS. 2022. Complete de novo assembly of *Wolbachia* endosymbiont of *Diaphorina citri* Kuwayama (Hemiptera: Liviidae) using long-read genome sequencing. 1. Sci Rep 12:125.
11. Sun J, Lu F, Luo Y, Bie L, Xu L, Wang Y. 2023. OrthoVenn3: an integrated platform for exploring and visualizing orthologous data across genomes. Nucleic Acids Research 51:W397–W403.
12. Meier-Kolthoff JP, Auch AF, Klenk H-P, Göker M. 2013. Genome sequence-based species delimitation with confidence intervals and improved distance functions. BMC Bioinformatics 14:60.
13. Baldo L, Lo N, Werren JH. 2005. Mosaic Nature of the *Wolbachia* Surface Protein. Journal of Bacteriology 187:5406–5418.
