# Supplementary material for: A dual insect symbiont and plant pathogen improves insect host fitness under arginine limitation
Source: mBio. 2025 Feb 25;16(4):e03588-24. doi: 10.1128/mbio.03588-24 (PMC11980576; doi:10.1128/mbio.03588-24)

Table S1. Illumina read statistics of metagenomic assembly.

	Run 1 (P)	Run 2 (S)	Unassigned
Input Read Pairs	227,889,247	150,454,908	35,683,995
Both Surviving	36,118,108 (15.85%)	57,002,622 (37.89%)	32,484,456 (91.03%)
Forward-Only Surviving	148,727,476 (65.26%)	84,419,171 (56.11%)	1,133,352 (3.18%)
Reverse-Only Surviving	1,106,417 (0.49%)	941,093 (0.63%)	477,762 (1.34%)
Dropped	41,937,246 (18.40%)	8,092,022 (5.38%)	1,588,425 (4.45%)

Table S2. BUSCO statistics based on data generated from the Prokka pipeline.

Assembly	Complete BUSCOs	Complete and Single-copy BUSCOs	Complete and Duplicated BUSCOs	Fragmented BUSCOs	Missing BUSCOs	Total BUSCO Groups
Carsonella-BC_CA	45	45	0	10	311	366
Carsonella-BC_WA (NZ_CP019943.1)	45	45	0	10	311	366
Carsonella-PV (ASM1036v1)	45	45	0	8	313	366
L. psyllaurous (de novo Assembly)	500	500	0	2	137	639
L. psyllaurous (Remapping Assembly)	492	492	0	4	143	639
L. psyllaurous (Reference NC_014774.1)	492	492	0	4	143	639
Wolbachia-BC-Bin1	201	177	24	16	147	364
Wolbachia-BC-Bin2	347	340	7	1	16	364
Wolbachia-DC (NZ_CP048820.1)	344	341	3	1	19	364

Table S3. Prokka annotations of symbiont metagenomes and reference genomes.

	Carsonella assemblies		*L. psyllaurous* assemblies			*Wolbachia* assemblies		
	Carsonella-BC-CA	*Carsonella*-BC-WA [1]	*de novo*	Remapping	Reference [2]	*Wolbachia*-BC-Bin1	*Wolbachia*-BC-Bin2	*Wolbachia*-DC [3]
Genes	231	231	1176	1143	1217	1543	1722	1493
CDS	201	201	1133	1089	1163	1513	1684	1454
tRNA	28	28	39	44	44	27	34	35
rRNA	2 (23S, 16S)	2 (23S, 16S)	3	9	9	3	3	3
tmRNA	0	0	1	1	1	0	1	1

Table S4. BLASTn hits comparing Carsonella 16s rRNA genes for biotyping. (a) Full length 16S rRNA sequences are included for *Carsonella-BC-WA*, *Carsonella- BC-CA*, and AF211126.2. Only partial 16S rRNA sequence is available for KR045612.1. (b) The 16S rRNA sequences were truncated to standardize the alignment to KR045612.1.

**a**	*Carsonella-BC_WA (NZ_CP019943.1)*	*Carsonella- BC_CA*	KR045612.1 (Northwestern)	AF211126.2 (Western)
Carsonella-BC_WA	-	-	-	-
Carsonella-BC_CA	(1512/1518; 99.6%)	-	-	-
KR045612.1 (Northwestern)	(921/930)	(916/930)	-	-
AF211126.2 (Western)	(1513/1518; 99.6%)	(1517/1518; 99.9%)	(917/930)	-

**b**	*Carsonella-BC-WA (NZ_CP019943.1)*	*Carsonella- BC-CA*	KR045612.1 (Northwestern)	AF211126.2 (Western)
Carsonella-BC_WA	-	-	-	-
Carsonella- BC_CA	(916/921; 99.46%)	-	-	-
KR045612.1 (Northwestern)	(921/930; 99.03%)	(916/930; 98.49%)	-	-
AF211126.2 (Western)	(917/921; 99.57%)	(920/921; 99.89%)	(917/930; 98.60%)	-

Table S5. Best BLASTx hits to the nr database for singletons identified in OrthoVenn3 for the A) de novo assembly (here) and B) *L. psyllaurous* reference.

Gene Name	Best Hit	Query Coverage	Percent Identity	E-value
**A**				
HJGOALFG_00123 Hypothetical Protein	*Candidatus* Liberibacter solanacearum Hypothetical protein	18%	92.31%	4.00E-04
HJGOALFG_00224 Hypothetical Protein	*Candidatus* Liberibacter solanacearum Hypothetical protein	64%	50.94%	2.00E-05
HJGOALFG_00466 Hypothetical Protein	*Candidatus* Liberibacter ctenarytainae type II toxin-antitoxin system HicB family antitoxin	70%	52.58%	2.00E-27
HJGOALFG_00591 Hypothetical Protein	*Candidatus* Liberibacter solanacearum Helix-turn-helix transcriptional regulator	99%	98.95%	1.00E-133
HJGOALFG_01124 Hypothetical Protein	*Candidatus* Liberibacter solanacearum Hypothetical protein	98%	97.18%	4.00E-42
HJGOALFG_01138 Hypothetical Protein	*Liberibacter* phage P-PA19-1	99%	92.17%	6.00E-73
**B**				
BGOIPHFH_00190 Hypothetical Protein	*Candidatus Liberibacter solanacearum* Hypothetical protein	99%	100.00%	3.00E-130
BGOIPHFH_00191 Hypothetical Protein	*Candidatus Liberibacter solanacearum* Hypothetical protein	98%	100.00%	5.00E-45
BGOIPHFH_00208 Hypothetical Protein	*Candidatus Liberibacter solanacearum* Anti-repressor protein	98%	98.41%	4.00E-36
BGOIPHFH_00216 Hypothetical Protein	*Candidatus Liberibacter solanacearum* Hypothetical protein	98%	100.00%	2.00E-33
BGOIPHFH_01125 Putative 3-methyladenine DNA glycosylase	*Candidatus Liberibacter solanacearum* DNA-3-methyladenine glycosylase	99%	100.00%	2.00E-144
BGOIPHFH_01126 Hypothetical Protein	*Candidatus Liberibacter solanacearum* Small effector protein	38%	100.00%	3.00E-09
BGOIPHFH_01127 Hypothetical Protein	*Candidatus Liberibacter solanacearum* Hypothetical protein	99%	99.14%	9.00E-54
BGOIPHFH_01171 Hypothetical Protein	*Candidatus Liberibacter solanacearum* Hypothetical protein	98%	98.70%	1.00E-48
BGOIPHFH_01179 Hypothetical Protein	*Candidatus Liberibacter solanacearum* Hypothetical protein	98%	100.00%	2.00E-33

Table S8. Total RNA-Seq reads sequenced, quality trimmed, and successfully mapped as pairs for each bacteriome sample in infected and uninfected *Bactericera cockerelli.*

RNA-Seq samples	Total reads	Total reads after trimming	Paired reads	Unpaired reads	Overall alignment rate
Infected					
Infected Bacteriome 1	30,664,667	30,531,388	19,273,770	11,257,618	69.37%
Infected Bacteriome 2	29,377,452	29,273,589	20,104,335	9,169,254	69.73%
Infected Bacteriome 3	29,907,661	29,794,805	19,665,923	10,128,882	62.73%
Infected Body Cells 1	39,934,237	39,770,301	25,540,217	14,230,084	67.03%
Infected Body Cells 2	31,490,915	31,380,967	20,663,143	10,717,824	69.91%
Infected Body Cells 3	30,122,253	30,028,498	21,891,983	8,136,515	65.85%
Uninfected					
Uninfected Bacteriome 1	29,064,126	28,964,905	20,112,044	8,852,861	67.76%
Uninfected Bacteriome 2	31,308,459	31,224,666	23,193,196	8,031,470	68.38%
Uninfected Bacteriome 3	31,160,749	31,035,840	20,360,748	10,675,092	68.32%
Uninfected Body Cells 1	30,868,768	30,766,057	21,111,672	9,654,385	65.92%
Uninfected Body Cells 2	26,048,179	25,954,428	16,859,480	9,094,948	65.09%
Uninfected Body Cells 3	36,104,921	35,978,502	24,600,049	11,378,453	70.19%

Table S10. **Differential expression data of symbiosis-related genes and HTGs of *B. cockerelli.*** All pairwise comparisons are included. Asterisk indicates significantly differentially expressed genes (FDR adjusted p value < 0.05 and fold change >1.5X)

Name	EC Number	Enzyme	*B. cockerelli gene ID*	Infected bacteriomes vs. Uninfected bacteriomes		
				LogFC	FDR	sig
Collaborative essential amino acid genes						
P5CS	2.7.2.11/1.2.1.41	delta-1-pyrroline-5-carboxylate synthetase	ANN07147	-0.148302999	0.682959832	
OAT	2.6.1.13	ornithine--oxo-acid transaminase	ANN07991	N/A	N/A	
BCAT	2.6.1.42	branched-chain amino acid aminotransferase	ANN10973	0.580175149	0.236994225	
SDS	4.3.1.17/4.3.1.19	L-serine/L-threonine ammonia-lyase	ANN22278	0.152254618	0.689734533	
AAT	2.6.1.1	Aspartate aminotransferase	ANN17803	0.345004554	0.311882105	
CBL-1	4.4.1.13	cysteine-S-conjugate beta-lyase	ANN16046	-0.489399826	0.017183392	
CBL-2	4.4.1.13	cysteine-S-conjugate beta-lyase	ANN16088	0.81729133	1.96371E-05	*
BHMT-1	2.1.1.10	homocysteine S-methyltransferase	ANN06058	-0.317290506	0.139566646	
BHMT-2	2.1.1.10	homocysteine S-methyltransferase	ANN06059	0.149228778	0.662484072	
Non-essential amino acid psyllid genes						
GOGAT	1.4.1.13	Glutamate synthase (NADH)	ANN19375	-0.086411817	0.775732184	
GDH-1	1.4.1.3	glutamate dehydrogenase	ANN04489	0.19392671	0.476585318	
GDH-2	1.4.1.3	glutamate dehydrogenase	ANN10746	1.231384348	0.315089593	
GS-1	6.3.1.2	glutamine synthetase	ANN10145	0.151396127	0.62172292	
GS-2	6.3.1.2	glutamine synthetase	ANN10148	0.120728267	0.704623314	
PAH	1.14.16.1	phenylalanine-4-hydroxylase	ANN09753	0.286245198	0.300160731	
ASNS-1	6.3.5.4	Asparagine synthase	ANN02335	-0.287725936	0.335061789	
ASNS-2	6.3.5.4	Asparagine synthase	ANN09881	-0.317122952	0.388246253	
ASNS-3	6.3.5.4	Asparagine synthase	ANN21723	-0.41369228	0.200274752	

ASPG	3.5.1.1	L-asparaginase	ANN07324	0.542316822	0.298698793	
P5CR	1.5.1.2	pyrroline-5-carboxylate reductase	ANN22280	0.026800489	0.950321559	
CBS	4.2.1.22	cystathionine beta-synthase	ANN22283	-0.220199292	0.32722157	
CGL-1	4.4.1.1	Cystathionine gamma lyase	ANN04485	0.23500436	0.30029288	
CGL-2	4.4.1.1	Cystathionine gamma lyase	ANN04499	N/A	N/A	
PGDH	1.1.1.95	Phosphoglycerate dehydrogenase	ANN19564	0.244801149	0.279318873	
PSAT	2.6.1.52	Phosphoserine aminotransferase	ANN12529	-0.009799131	0.9873677	
PSP	3.1.3.3	Phosphoserine phosphatase	ANN22282	-0.031993662	0.944965715	
Horizontally transferred genes						
ASL-1	4.3.2.1	Argininosuccinate lyase	ANN12874	0.025920348	0.955294249	
ASL-2a	4.3.2.1	Argininosuccinate lyase	ANN10361	0.27114067	0.289890166	
ASL-2b	4.3.2.1	Argininosuccinate lyase	ANN20354	-0.678679582	0.000212438	*
CM-1	5.4.99.5	Chorismate mutase	ANN05927	1.104558054	0.040151722	*
CM-2	5.4.99.5	Chorismate mutase	ANN06704	-0.602756302	0.012809467	*
CM-3	5.4.99.5	Chorismate mutase	ANN17115	0.748492572	0.007109153	*
MUTY	3.2.2.31	A/G-specific adenine glycosylase	ANN05978	N/A	N/A	
ORF-1	N/A	AAA-ATPase-like	ANN01458	-0.47451099	0.00651424	
ORF-2	N/A	AAA-ATPase-like	ANN17655	0.029456545	0.939250348	
ORF-3a	N/A	AAA-ATPase-like	ANN18155	0.348178888	0.085654272	
ORF-3b	N/A	AAA-ATPase-like	ANN18146	0.499525833	0.046711265	
RIBC	2.5.1.9	Riboflavin synthase	ANN14810	-0.343764217	0.209294232	
RSMJ	2.1.1.242	16S rRNA methyltransferase	ANN13802	0.373916073	0.062149567	
YDCJ	N/A	VOC family protein	ANN12599	0.169043101	0.575095606	

Table S12. Accession numbers of *Wolbachia* proteins, 16S rRNA, *wsp* sequences obtained from NCBI

Abbreviated name	RefSeq Genome Accession
B_C_megacephala	NZ_CP021120.1
B_L_clavipes	NZ_QJHA01000026.1
B_nVitB_N_vitripennis	NZ_GL883637.1
B_wAlbB_A_albopictus	NZ_CP101657.1
B_wBta_B_tabaci	NZ_CP016430.1
B_wLug_N_lugens	NZ_MUIY01000001.1
B_wTpre_T_pretiosum	NZ_CM003641.1
B_wDcitri_Dcitri	NZ_CP048820.1

wsp

host species	GenBank Accession
B. cockerelli isolate Orange1	AY971925
B. cockerelli isolate Orange8	AY971932
B. cockerelli Nebraska1	AY971945
B. cockerelli Nebraska2	AY971946

Table S13. Concentrations of artificial diet components used for the two diets (with arginine and without arginine)

Nutrients	Molar Concentrations		Grams Per 400mL	
**Sugars**	With ARG	Without ARG	With ARG	Without ARG
Sucrose	0.5 M	0.5 M	68.4g	68.4g
**Essential Amino Acids**				
L-Arginine HCL	12.5 mM	0 M	1.0537g	0g
L-Histidine (Free Base)	7.5 mM	7.5 mM	0.4656g	0.4656g
L-Isoluecine (Allo Free)	7.5 mM	7.5 mM	0.3936g	0.3936g
L-Leucine HCL	7.5 mM	7.5 mM	0.3936g	0.3936g
L-Lysine HCl	7.5 mM	7.5 mM	0.548g	0.548g
L-Methionine	2.5 mM	2.5 mM	0.1495g	0.1495g
L-Phenylalanine	2.5 mM	2.5 mM	0.1653g	0.1653g
L-Threonine (Allo Free)	7.5 mM	7.5 mM	0.3574g	0.3574g
L-Tryptophane	2.5 mM	2.5 mM	0.2044g	0.2044g
L-Valine	7.5 mM	7.5 mM	0.3512g	0.3512g
**Total essential amino acids**	65 mM	52.5 mM		
**Nonessential Amino Acids**				
L-Alanine	5 mM	5 mM	0.178g	0.178g
L-Asparagine (Anhydrous)	12.5 mM	12.5 mM	0.6606g	0.6606g
L-Aspartic acid C.P.	12.5 mM	12.5 mM	0.6657g	0.6657g
L- Cysteine HCl monohydrate	2.5 mM	2.5 mM	0.1576g	0.1576g
L-Cystine	0.2 mM	0.2 mM	0.02g	0.02g
Gamma amino butyric acid	2 mM	2 mM	0.08g	0.08g
L-Glutamic acid	7.5 mM	20 mM	0.4413g	2.9424g
L-Glutamine (Commercial)	15 mM	15 mM	0.8769g	0.8769g
Glycine	1 mM	1 mM	0.03g	0.03g
L-Proline	5 mM	5 mM	0.2304g	0.2304g
L-Serine	5 mM	5 mM	0.21g	0.21g
L-Tyrosine	0.5 mM	0.5 mM	0.036g	0.036g
**Total nonessential amino acids**	**68.7 mM**	**81.2 mM**		
**Total Amino Acids**	**133.7 mM**	**133.7 mM**		
**Vitamins**				
p-Aminobenzoic acid	0.73 mM	0.73 mM	0.04g	0.04g
Ascorbic acid	5.68 mM	5.68 mM	0.4g	0.4g
Biotin	6 uM	6 uM	0.0004g	0.0004g
Calcium pantothenate	0.21 mM	0.21 mM	0.04g	0.04g
Folic acid	22 uM	22 uM	0.004g	0.004g

i-Inositol	1.39 mM	1.39 mM	0.1001g	0.1001g
Nicotinic acid	0.812 mM	0.812 mM	0.04g	0.04g
Pyridoxine HCl	0.121 mM	0.121 mM	0.01g	0.01g
Thiamine HCl	74 uM	74 uM	0.01g	0.01g
**Salts, Buffers, and Sterol**				
Calcium citrate Tetrahydrate	0.175 mM	0.175 mM	0.04g	0.04g
Cholesterol benzoate	50 uM	50 uM	0.01g	0.01g
Potassium phosphate monobasic	18.37 mM	18.37 mM	1g	1g
Sodium chloride	0.217 mM	0.217 mM	0.0508g	0.0508g
Cupric chloride	14 uM	14 uM	0.001g	0.001g
Ferric chloride hexahydrate	49 uM	49 uM	0.0053g	0.0053g
Manganese (II) chloride Tetrahydrate	101 uM	101 uM	0.0032g	0.0032g
Zinc sulfate Monohydrate	30 uM	30 uM	0.0021g	0.0021g
Magnesium chloride-6H2O	9.837 mM	9.837 mM	0.8g	0.8g
Choline chloride	3.579 mM	3.579 mM	0.2g	0.2g

Figure S1. Venn diagram showing orthologous clusters among *Wolbachia* strains. The shared and unique orthologous clusters among the *Wolbachia*-Bin1, *Wolbachia*-Bin2 and *Wolbachia*-DC genome assembles were identified using OrthoVenn3.

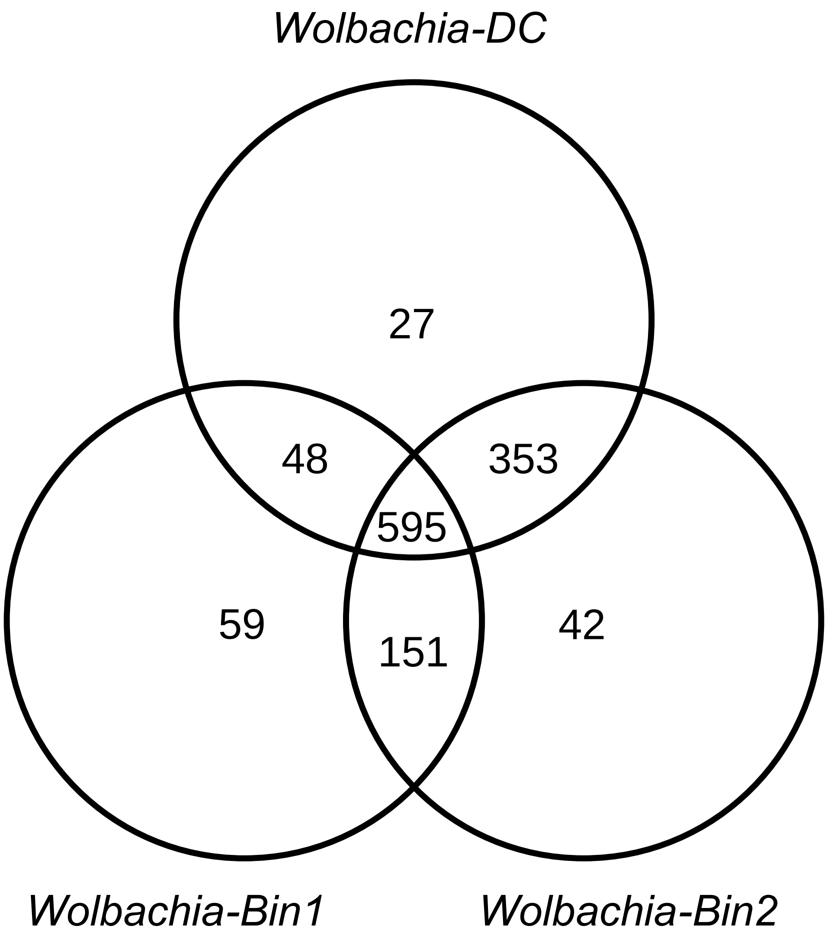

Figure S2. Phylogenetic trees of two *Wolbachia* strains, Bin1 and Bin2, for (A) *wsp*, (B) 16S rRNA gene, and (C) single-copy orthologs. Abbreviations indicate which insect species *Wolbachia* was sequenced from. *Nilaparvata lugens* (*Nlug*); *Leptopilina clavipes* (*Lcal*), *Nasonia vitripennis* (*Nvit*), *Trichogramma pretisoum* (*Tpre*), *Chrysomya megacephala* (*Cmeg*), *Bemesia tabaci* (*Btab*), *D. citri* (*DC*), *Aedes albopictus* (*Aalb*), *Bactericera maculipennis* (*Bmac*), *B. cockerelli*-northwestern (*BC-NW*), Western (W) and Central (C) haplotypes. Accession numbers used for phylogenetic analyses are found in **Table S12**.

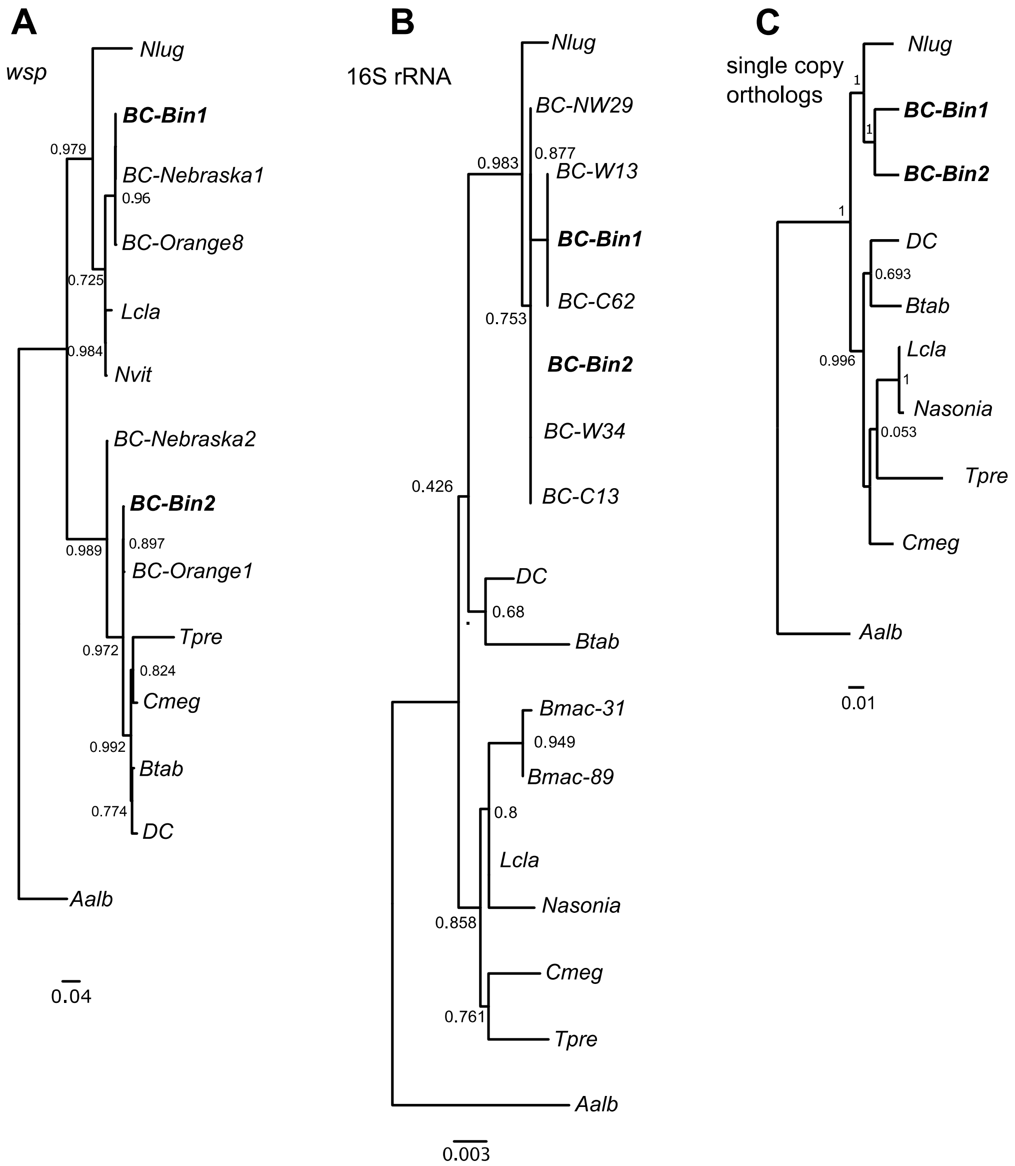

Figure S3. Phylogenetic analysis of *Wolbachia* strains co-infecting *B. cockerelli* and related species, showing gene family expansions and contractions over evolutionary time. Divergence times are shown on a timeline in million years ago (Mya). Pie charts at each node depict the gene family expansions (purple) and contractions (blue) identified by Cafe5 analysis, with numbers indicating the net gains (+) or losses (−) of gene families. This analysis suggests significant diversification within *Wolbachia* strains, including the co-infecting strains Bin1 and Bin2, which diverged around 1 million years ago.

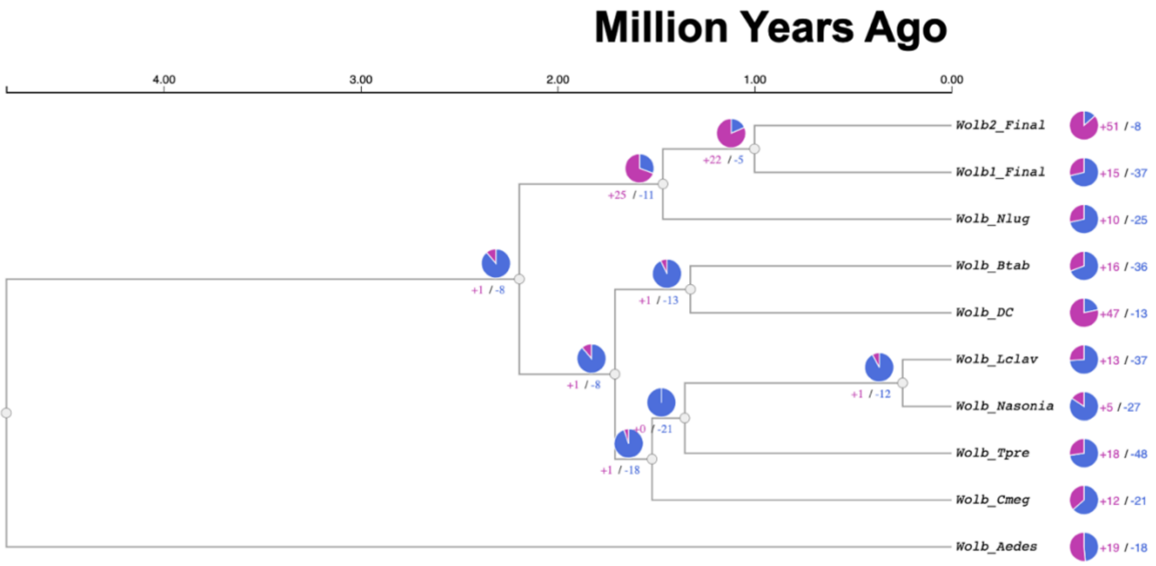

Supplement: Document S2 — Tables S1-S5, S8, S10, S12, and S13 and Figures S1-S3. [file mbio.03588-24-s0002.pdf]
