# Supplementary material for: A dual insect symbiont and plant pathogen improves insect host fitness under arginine limitation
Source: mBio. 2025 Feb 25;16(4):e03588-24. doi: 10.1128/mbio.03588-24 (PMC11980576; doi:10.1128/mbio.03588-24)
Supplement: Document S3 — Supplemental methods. [file mbio.03588-24-s0003.pdf]

Isofemale line establishment

Fifth instar psyllids were collected and put on 3-week-old healthy, uninfected tomato plants. Newly emerged one-day old adults were collected, sexed, and segregated by sex into female-only and male-only mesh cages, each with 3-week-old healthy, uninfected tomato plants. The reason why psyllids were sexed and separated on day one of the adult stage was to ensure that females were not inseminated multiple times before fitness trials began given that reproductive maturity of psyllids can begin 48hr post-adult molt depending on sex (1). Adult female and male psyllids were allowed to mature in their separate cages for 5 days given the pre-mating period (sexual maturity) can take up to 4.2 ± 0.5 days in *B. cockerelli* (2). After five days, one virgin female and one virgin male were paired in a vial for 6 hours for mating. Each mated female was then placed on its own healthy, uninfected 3-week-old tomato plant, and the plant was placed inside a mesh cage to contain the mated female and subsequent isofemale line. The mated female oviposited on its plant for 5 days and then was removed from its plant.

Real-time quantitative PCR (qPCR)

Diagnostic qPCR was performed to confirm the presence or absence of *L. psyllaurous* in both the infected and uninfected psyllid lines before and after trials. The qPCR detection reactions were run with three technical replicates using the Y DRAG Q-PCR- IGS-7F/7R primers designed in Casteel et al. (2012) (3) and iTaq Universal SYBR Green Supermix on a Bio-Rad CFX96 touch machine (Bio-Rad, Hercules, CA, USA). The qPCR conditions were 95°C for 5 mins, followed by 40 cycles of 95°C for 30 sec, 60°C for 30 sec, and the final melt curve for primer specificity from 65°C to 95°C with 0.5 °C.

Absolute quantification was performed to measure symbiont genome copy number relative to insect genome copy number within bacteriomes compared to the body tissues. Similar to Kwak and Hansen (2023) (4), fifth instar nymphs of both *L. psyllaurous*-infected and -uninfected *B. cockerelli* were collected for bacteriome dissections. Bacteriomes and the rest of the body tissues without the bacteriomes were pooled from 15 individuals of both sexes with three biological replicates. Genomic DNA was extracted using Quick-DNA Microprep Plus Kit (Zymo, Irvine, CA, USA), RNase A (Thermo Scientific, Waltham, MA, USA) treated, and cleaned with the Genomic DNA Clean and Concentrator kit-10 (Zymo research, Irvine, CA, USA). We targeted the *L. psyllaurous* 16S-23S IGS region using Y-DRAG (see above), *Wolbachia*-Bin1 and Bin2 surface protein (wsp), and the *B. cockerelli* FORKHEAD genes using the same primers in Kwak and Hansen (2023) (4) and here; the forward primer for *Wolbachia*-Bin1 *wsp* was designed here (*wsp*_Bin1F: 5'-GCATCTTTTCTCGCTGGAGG-3') and paired with the reverse primer that binds to both *Wolbachia*-Bin1 and Bin2 (4). The PCR products were cloned into the pGEM-T Easy vector (Promega) and amplified in *Escherichia coli* JM109. Plasmids with inserts were amplified in *E. coli* and purified using Plasmid Minikit (Invitrogen). Subsequently, Plasmid DNA was quantified using a Qubit 4.0 Fluorometer (Invitrogen, Carlsbad, CA, USA). Copy numbers of the plasmid DNA were calculated based on their concentration and molecular weights. For each target gene, 10^8, 10^7, 10^6, 10^5, 10^4 and 10^3 copies/mL of plasmid DNA solutions were freshly prepared for standard samples. The qPCR reactions were the same as the diagnostic qPCR above.

Symbiont filtration and DNA extraction

Symbiont cells of *L. psyllaurous* infected *B. cockerelli* were filtered from 0.3 g of mixed age psyllids from both sexes. Filtration was done according to the study by Hansen and Moran (2012) (5) with modifications as follows. First, buffer A (6) was used for the pellet portion (referred here as P sample; which primarily contains *Carsonella* cells) and 1X PBS was used for the supernatant portion of the following protocol (referred here as S sample; which primarily contains *L. psyllaurous* and *Wolbachia* cells). Second, after the 1000 rpm centrifugation step, both the pellet and supernatant samples were used for subsequent filtration steps. After the last centrifugation step, DNA was extracted with DNeasy Blood &

Tissue Kit (QIAGEN) and treated with RNase A. DNA quality and quantity was checked using SpectraMax QuickDrop Micro-Volume Spectrophotometer (Molecular Devices) and Qubit 4.0 Fluorometer (Invitrogen). The samples P and S were sequenced in separate libraries within the same lane for metagenomic sequencing (see methods details below).

Metagenomic sequencing and endosymbiont genome assembly

The library for P and S samples (see above) was sequenced as paired-end, 300 bp reads on one lane of NextSeq2000 using the Illumina P2 reaction kit. To filter out insect host reads from P and S samples (see above), reads were aligned with bowtie2 v2.4.5 (7) to the insect genome, *B. cockerelli* (NCBI Accession number: GCA_024516035.1 (8). Reads that did not align to *B. cockerelli's* genome were further assembled into contigs using MEGAHIT (9) for the *Carsonella*, *L. psyllaurous* and *Wolbachia* assemblies.

Carsonella

To identify *Carsonella* contigs BLASTn was conducted against the *Ca.* Carsonella ruddii *-BC* reference genome (NZ_CP019943.1) (10). All reads that were identified from *Carsonella* candidate contigs were then reassembled *de novo* using MEGAHIT (9) and examined in Mauve (11) against the reference genome (NZ_CP019943.1) (10) to observe genome synteny. The percent nucleotide similarity between the new closed *Carsonella* assembly, *Carsonella*-BC-CA, and the reference (NZ_CP019943.1 (10) was conducted using JSpeciesWS (12). Draft genome completeness and quality were examined by computing the N50 using QUAST (13), and by conducting BUSCO v5.4.4 (14) and Mauve (11) using the reference genome NZ_CP019943.1 (10) and the *Carsonella* Hackberry psyllid reference assembly (AP009180.1) (15). Further, Prokka v1.14.5 (16) was used to annotate both the reference genome NZ_CP019943.1 (10) and the *Carsonella*-BC-CA assembly and gene identity and order were compared. Genotyping of *Carsonella* strains was conducted by using the 16S rRNA sequences using BLASTn (17).

Liberibacter psyllaurous

To identify *L. psyllaurous* contigs from MEGAHIT (9), a similar pipeline was conducted as above for the *de novo Carsonella* assembly with the following modifications: *Carsonella* reads were filtered from contigs by aligning reads to the *Ca.* Carsonella ruddii *-BC* reference genome (NZ_CP019943.1) (10). Remaining contigs were then blasted to the reference *Ca*. L. psyllaurous genome (NC_014774.1) (18) using BLASTn. Reads from these latter contigs were then further binned using Metabat2 v2.15 (19) to further filter out *Wolbachia* and *Carsonella* reads using BLASTn. Next, reads were reassembled a second time *de novo* using MEGAHIT (9). Assembly quality and completeness analyses were conducted similar to above. The percent nucleotide similarity between the reference genome NC_014774.1 (18) and the *L. psyllaurous* assembly here was conducted as above using JSpeciesWS (12). A remapping consensus sequence approach was also conducted in addition to the *de novo* assembly to aid in gene annotations including the verification of rRNA gene copies. For the remapping approach all metagenomic reads were aligned to the reference genome using bowtie2 v2.4.5 (7). Bcftools (20) and seqtk (21) was used to generate a consensus sequence from the assembly of metagenomic reads mapped to the reference genome. OrthoVenn3 (22) and tBLASTx was used to identify gene copies and unique genes present only in the new *de novo* assembly compared to the reference genome (NC_014774.1) (18). BLASTx (Updated to 2024/02/20) was then used to characterize these unique genes.

Wolbachia

For the *Wolbachia* assembly, contaminant reads were removed similar to above. In addition, raw PacBio reads and Illumina reads from the *B. cockerelli* genome sequencing project (SRR18584432-SRR18584429) (8), which are from the same psyllid line from our laboratory, were filtered for contaminant reads and then assembled together with the filtered Illumina metagenomic reads (here) to generate a short and long-read hybrid assembly using hybridSpades v3.15.4 (23, 24). All contigs 5,000bp and above were taken and binned using Anvio v8.0 (25) with manual curation guided by PFAM (v33.1) and TIGRFAM (v15.0) databases. Assembly quality and completeness analyses were conducted similar to above. Given two *Wolbachia* strains were expected to co-infect psyllid individuals (26), BLASTn was conducted on *wsp* and 16S rRNA gene sequences and pairwise DNA-DNA hybridization (DDH) estimates were calculated between the two putative *Wolbachia* assemblies using the Genome-to-Genome Distance Calculator 3.0 (GGDC2) (27). For the 16S rRNA gene, reads were analyzed through a remapping approach similar to above and visualized in Tablet (28) to help determine strain type. Gene annotations were determined using the same annotation pipeline above. OrthoVenn3 (22) was used to find orthologous gene clusters and singletons between *Wolbachia* genome assemblies here and the *Wolbachia* genome assembly from the psyllid *Diaphorina citri* (referred to hereafter as *Wolbachia*-DC) (NCBI Accession Number: NZ_CP048820.1). Singletons were further blasted against the NCBI nr database with BLASTx to help determine gene annotations.

Phylogenetic analyses

Phylogenetic analyses of the *wsp* and 16S rRNA genes for the two *Wolbachia* strains assembled here were conducted by first aligning gene sequences with MUSCLE v3.8.1551 (29). Aligned sequences were then subjected to approximately-maximum-likelihood tree estimation with FastTree v2.1.11 (30). The nucleotide alignments were analyzed with a generalized time-reversible model of evolution under a discrete gamma model with 20 rate categories and SH-like local supports are reported. Phylogenetic analyses with single-copy orthologs were conducted with OrthoVenn3 (22), which uses FastTree v2.1.11 (30) using default parameters. The expansions and contractions analysis were also conducted in OrthoVenn3 (22), which uses Cafe5 (31) using default parameters and using auto search for divergence times using TimeTree5 (32). Outgroup Accession numbers used for phylogenetic analyses are found in **Table S12**.

Bacteriome dissections and total RNA extraction

For both *L. psyllaurous* uninfected (CA) and infected (CA-Lpsy) lines, sixty bacteriomes and the rest of the body, without the bacteriomes, were dissected from 5th instar nymphs and pooled into two separate tissue samples per biological replicate similar to Pers & Hansen (2021) (33). A total of three biological replicate samples of bacteriomes and body tissues from the uninfected line and three biological replicate samples of bacteriomes and body tissues from the infected line were obtained resulting in 12 samples total. The developmental stage of *B. cockerelli* nymphs was determined based on their physical characteristics and growth periods as described in Knowlton & Janes (1931) (34). Samples were stored at -80 °C in the RNAprotect bacterial reagent (QIAGEN, Germantown, MD, USA) until RNA extraction. Diagnostic qPCR with *Y-DRAG* primers was conducted (see methods above) to ensure infected psyllids were infected and un-infected psyllids were un-infected before and after trials.

Total RNA was extracted from the pooled bacteriome and corresponding body tissue samples using the Quick-RNA Microprep kit and RNA Clean & Concentrator kit-5 (Zymo Research, Irvine, CA, USA). The quality and quantity of the purified RNA samples were assessed using the Agilent TapeStation (Agilent, Santa Clara, CA, USA) and Qubit 4.0 Fluorometer (Invitrogen, Carlsbad, CA, USA) at the Institute of Integrative Genome Biology Instrumentation Facilities Services at the University of California, Riverside.

RNA sequencing and bioinformatic analysis

Total RNA for samples were sent to the DNA Technology Core at the University of California, Davis for library preparation and sequencing. Strand-specific and barcode indexed RNA-seq libraries were generated from 300 ng of total RNA for each sample using Kapa mRNA Stranded library preparation kit (KK8421, Kapa Biosystems, Cape Town, South Africa), followed by 12 cycles of PCR amplification. The fragment size distribution of the libraries was verified via micro-capillary gel electrophoresis on a Bioanalyzer 2100 (Agilent, Santa Clara, CA). The libraries were quantified by fluorometry on a Qubit fluorometer (LifeTechnologies, Carlsbad, CA) and pooled in equimolar ratios. The pool was quantified by qPCR with a Kapa Library Quant kit (Kapa Biosystems) and sequenced on one lane of the Illumina NovaSeq S4 platform (Illumina, San Diego, CA) with paired-end 150bp reads.

Following the same RNAseq pipeline detailed in previous studies (4, 33, 35), raw RNA-seq reads were quality checked and trimmed with FASTQC v.0.11.8 (36) and Trimmomatic v. 0. 36 (37) with the following parameters: ILLUMINACLIP:TruSeq3-PE.fa:2:30:10 LEADING:3 TRAILING:3 SLIDINGWINDOW:4:15 MINLEN:36. The trimmed reads were aligned using HISAT v. 2.1. 0 (38) against the chromosomal assembly of *B. cockerelli*-isoF-IL for NCBI Accession number: GCA_024516035.1 (8). The mapped reads for each gene were quantified as raw read counts using StringTie v.2.2.1 (39) using gff files annotated for *B. cockerelli* (8).

Differentially expressed genes between uninfected and infected tissues were determined with the exact test with EdgeR (40) in R version 4.2.0 (41). Statistical significance of differentially expressed genes was determined with a false discovery rate (FDR) adjusted $P \leq 0.05$ and ≥ 1.5-fold change of the normalized expression values, where "logFC" indicate log2 fold change between the groups as described in Argandona et al. (2023) (35) and Kwak and Hansen (2023) (4). Annotations for subsets of genes that are related to horizontally transferred genes (HTGs) and symbiosis genes were obtained from the previous genome analyses (8).

Feeding chamber set-up for artificial diet bioassay

A small culture dish (35 by 10 mm, nontreated polystyrene, 430588, corning life science) was used as a feeding chamber by cutting the bottom lid. A 2.5 by 2.5 cm piece of parafilm was stretched over the cut Petri dish lid, and 800 uL of artificial diet was pipetted onto this depression. A second parafilm strip was stretched over the first to seal the artificial diet, and the perimeter of the Petri dish was sealed with parafilm to prevent leakage. A tight seal was important to prevent the diet from leaking out of the well and to ensure that the psyllids could pierce their stylets through the film. Five 3rd instar psyllids were transferred into each rearing arena and then covered by inverting the second plate over the first, trapping the psyllids in the feeding chamber. Two Petri dishes were then sealed together with another strip of Parafilm to prevent psyllids from escaping and desiccation. The psyllids were allowed to develop in these arenas for until they molted into an adult. A fresh diet solution and new parafilm coverings were provided every 24 hours to prevent excess humidity and mold growth. The feeding chamber was modified by Hall et al., (2010) (42).

The feeding chambers were then placed in a growth chamber set at 25°C, 40% relative humidity, and a photoperiod of 18:6 (L:D) hours, with light intensity at 4200 lux. Data was collected daily to monitor the survival of the nymphs as they emerged into adults. As the psyllids molted into adults, each adult that survived was then weighed within 24hr of molting using a New Classic MF Semi-micro Analytical balance (Metter Toledo, LLC, Columbus, OH, USA) and their mass was recorded in mg. Weight is commonly used as a surrogate fitness measurement for insects (43).

Statistical analysis of artificial diet bioassay

The dependent variables compared between *L. psyllaurous*-infected and uninfected psyllids on the two different diet treatments were nymphal development time in days (the duration it took for the 3rd instar to the emergence of the adult stage) and the weight of the adults 24hr after molting. These same two dependent variables were also compared between the two different diet treatments within each

psyllid *L. psyllaurous* infection treatment. For both pairwise comparisons a one-way ANOVA using IBM SPSS statistics v29.0.2.0 (44) was used and statistical significance was $p \leq 0.05$.

Reference

1. Guédot C, Horton DR, Landolt PJ. 2012. Age at reproductive maturity and effect of age and time of day on sex attraction in the potato psyllid *Bactericera cockerelli*. Insect Science 19:585–594.

2. Abdullah NM. 2008. Life history of the potato psyllid *Bactericera cockerelli* (Homoptera: Psyllidae) in controlled environment agriculture in Arizona. African Journal of Agricultural Research.

3. Casteel CL, Hansen AK, Walling LL, Paine TD. 2012. Manipulation of Plant Defense Responses by the Tomato Psyllid (*Bactericerca cockerelli*) and Its Associated Endosymbiont *Candidatus* Liberibacter Psyllaurous. PLOS ONE 7:e35191.

4. Kwak Y, Hansen AK. 2023. Unveiling metabolic integration in psyllids and their nutritional endosymbionts through comparative transcriptomics analysis. iScience 26:107930.

5. Hansen AK, Moran NA. 2012. Altered tRNA characteristics and 3′ maturation in bacterial symbionts with reduced genomes. Nucleic Acids Research 40:7870.

6. Hansen AK, Moran NA. 2011. Aphid genome expression reveals host–symbiont cooperation in the production of amino acids. Proceedings of the National Academy of Sciences 108:2849–2854.

7. Langmead B, Salzberg SL. 2012. Fast gapped-read alignment with Bowtie 2. Nat Methods 9:357–359.

8. Kwak Y, Argandona JA, Degnan PH, Hansen AK. 2023. Chromosomal-level assembly of *Bactericera cockerelli* reveals rampant gene family expansions impacting genome structure, function and insect-microbe-plant-interactions. Molecular Ecology Resources 23:233–252.

9. Li D, Liu C-M, Luo R, Sadakane K, Lam T-W. 2015. MEGAHIT: an ultra-fast single-node solution for large and complex metagenomics assembly via succinct de Bruijn graph. Bioinformatics 31:1674–1676.

10. Riley AB, Kim D, Hansen AK. 2017. Genome Sequence of "*Candidatus* Carsonella ruddii" Strain BC, a Nutritional Endosymbiont of *Bactericera cockerelli*. Genome Announcements 5:10.1128/genomea.00236-17.

11. Darling ACE, Mau B, Blattner FR, Perna NT. 2004. Mauve: Multiple Alignment of Conserved Genomic Sequence With Rearrangements. Genome Res 14:1394–1403.

12. Richter M, Rosselló-Móra R, Oliver Glöckner F, Peplies J. 2016. JSpeciesWS: a web server for prokaryotic species circumscription based on pairwise genome comparison. Bioinformatics 32:929–931.

13. Gurevich A, Saveliev V, Vyahhi N, Tesler G. 2013. QUAST: quality assessment tool for genome assemblies. Bioinformatics 29:1072–1075.

14. Simão FA, Waterhouse RM, Ioannidis P, Kriventseva EV, Zdobnov EM. 2015. BUSCO: assessing genome assembly and annotation completeness with single-copy orthologs. Bioinformatics 31:3210–3212.

15. Nakabachi A, Yamashita A, Toh H, Ishikawa H, Dunbar HE, Moran NA, Hattori M. 2006. The 160-Kilobase Genome of the Bacterial Endosymbiont *Carsonella*. Science 314:267–267.

16. Seemann T. 2014. Prokka: rapid prokaryotic genome annotation. Bioinformatics 30:2068–2069.

17. Zhang Z, Schwartz S, Wagner L, Miller W. 2000. A Greedy Algorithm for Aligning DNA Sequences. Journal of Computational Biology 7:203–214.

18. Lin H, Lou B, Glynn JM, Doddapaneni H, Civerolo EL, Chen C, Duan Y, Zhou L, Vahling CM. 2011. The Complete Genome Sequence of '*Candidatus* Liberibacter solanacearum', the Bacterium Associated with Potato Zebra Chip Disease. PLOS ONE 6:e19135.

19. Kang DD, Li F, Kirton E, Thomas A, Egan R, An H, Wang Z. 2019. MetaBAT 2: an adaptive binning algorithm for robust and efficient genome reconstruction from metagenome assemblies. PeerJ 7:e7359.

20. Li H. 2011. A statistical framework for SNP calling, mutation discovery, association mapping and population genetical parameter estimation from sequencing data. Bioinformatics 27:2987–2993.

21. Li H. 2024. Seqtk: Toolkit for Processing Sequences in FASTA/q Formats. C.

22. Sun J, Lu F, Luo Y, Bie L, Xu L, Wang Y. 2023. OrthoVenn3: an integrated platform for exploring and visualizing orthologous data across genomes. Nucleic Acids Research 51:W397–W403.

23. Prjibelski A, Antipov D, Meleshko D, Lapidus A, Korobeynikov A. 2020. Using SPAdes De Novo Assembler. Current Protocols in Bioinformatics 70:e102.

24. Antipov D, Korobeynikov A, McLean JS, Pevzner PA. 2016. hybridSPAdes: an algorithm for hybrid assembly of short and long reads. Bioinformatics 32:1009–1015.

25. Eren AM, Kiefl E, Shaiber A, Veseli I, Miller SE, Schechter MS, Fink I, Pan JN, Yousef M, Fogarty EC, Trigodet F, Watson AR, Esen ÖC, Moore RM, Clayssen Q, Lee MD, Kivenson V, Graham ED, Merrill BD, Karkman A, Blankenberg D, Eppley JM, Sjödin A, Scott JJ, Vázquez-Campos X, McKay LJ, McDaniel EA, Stevens SLR, Anderson RE, Fuessel J, Fernandez-Guerra A, Maignien L, Delmont TO, Willis AD. 2021. Community-led, integrated, reproducible multi-omics with anvi'o. Nat Microbiol 6:3–6.

26. Liu D, Trumble JT, Stouthamer R. 2006. Genetic differentiation between eastern populations and recent introductions of potato psyllid (*Bactericera cockerelli*) into western North America. Entomologia Experimentalis et Applicata 118:177–183.

27. Meier-Kolthoff JP, Auch AF, Klenk H-P, Göker M. 2013. Genome sequence-based species delimitation with confidence intervals and improved distance functions. BMC Bioinformatics 14:60.

28. Milne I, Stephen G, Bayer M, Cock PJA, Pritchard L, Cardle L, Shaw PD, Marshall D. 2013. Using Tablet for visual exploration of second-generation sequencing data. Briefings in Bioinformatics 14:193–202.

29. Edgar RC. 2004. MUSCLE: multiple sequence alignment with high accuracy and high throughput. Nucleic Acids Res 32:1792–1797.

30. Price MN, Dehal PS, Arkin AP. 2010. FastTree 2 – Approximately Maximum-Likelihood Trees for Large Alignments. PLOS ONE 5:e9490.

31. Mendes FK, Vanderpool D, Fulton B, Hahn MW. 2020. CAFE 5 models variation in evolutionary rates among gene families. Bioinformatics 36:5516–5518.

32. Kumar S, Suleski M, Craig JM, Kasprowicz AE, Sanderford M, Li M, Stecher G, Hedges SB. 2022. TimeTree 5: An Expanded Resource for Species Divergence Times. Molecular Biology and Evolution 39:msac174.

33. Pers D, Hansen AK. 2021. The boom and bust of the aphid’s essential amino acid metabolism across nymphal development. G3 Genes|Genomes|Genetics 11:jkab115.

34. Knowlton GF, Janes MJ. 1931. Studies on the Biology of *Paratrioza Cockerelli* (Sulc).*. Annals of the Entomological Society of America 24:283–292.

35. Argandona JA, Kim D, Hansen AK. 2023. Comparative transcriptomics of aphid species that diverged > 22 MYA reveals genes that are important for the maintenance of their symbiosis. 1. Sci Rep 13:5341.

36. Andrews. 2010. FastQC: A Quality Control Tool for High Throughput Sequence Dataics - FastQC A Quality Control tool for High Throughput Sequence Data. https://www.bioinformatics.babraham.ac.uk/projects/fastqc/. Retrieved 4 April 2022.

37. Bolger AM, Lohse M, Usadel B. 2014. Trimmomatic: a flexible trimmer for Illumina sequence data. Bioinformatics 30:2114–2120.

38. Pertea M, Kim D, Pertea GM, Leek JT, Salzberg SL. 2016. Transcript-level expression analysis of RNA-seq experiments with HISAT, StringTie and Ballgown. 9. Nat Protoc 11:1650–1667.

39. Pertea M, Pertea GM, Antonescu CM, Chang T-C, Mendell JT, Salzberg SL. 2015. StringTie enables improved reconstruction of a transcriptome from RNA-seq reads. 3. Nat Biotechnol 33:290–295.

40. Robinson MD, McCarthy DJ, Smyth GK. 2010. edgeR: a Bioconductor package for differential expression analysis of digital gene expression data. Bioinformatics 26:139–140.

41. R Core Team. 2020. R: A language and environment for statistical computing. R Foundation for Statistical Computing, Vienna, Austria. https://www.r-project.org/. Retrieved 4 April 2022.

42. Hall DG, Shatters RG, Carpenter JE, Shapiro JP. 2010. Research Toward an Artificial Diet for Adult Asian Citrus Psyllid. Annals of the Entomological Society of America 103:611–617.

43. Vogel KJ, Moran NA. 2011. Sources of variation in dietary requirements in an obligate nutritional symbiosis. Proc R Soc B 278:115–121.

44. SPSS Statistics 29.0.0. https://www.ibm.com/docs/en/spss-statistics/www.ibm.com/docs/en/spss-statistics/29.0.0. Retrieved 10 June 2024.
